# Polyploidization is accompanied by synonymous codon usage bias in the chloroplast genomes of both cotton and wheat

**DOI:** 10.1371/journal.pone.0242624

**Published:** 2020-11-19

**Authors:** Geng Tian, Guoqing Li, Yanling Liu, Qinghua Liu, Yanxia Wang, Guangmin Xia, Mengcheng Wang

**Affiliations:** 1 The Key Laboratory of Plant Development and Environmental Adaption, Ministry of Education, School of Life Science, Shandong University, Jinan, Shandong, China; 2 Shijiazhuang Academy of Agriculture and Forestry Sciences, Shijiazhuang, China; Institute for Biological Research "S. Stanković", University of Belgrade, SERBIA

## Abstract

Synonymous codon usage bias (SCUB) of both nuclear and organellar genes can mirror the evolutionary specialization of plants. The polyploidization process exposes the nucleus to genomic shock, a syndrome which promotes, among other genetic variants, SCUB. Its effect on organellar genes has not, however, been widely addressed. The present analysis targeted the chloroplast genomes of two leading polyploid crop species, namely cotton and bread wheat. The frequency of codons in the chloroplast genomes ending in either adenosine (NNA) or thymine (NNT) proved to be higher than those ending in either guanidine or cytosine (NNG or NNC), and this difference was conserved when comparisons were made between polyploid and diploid forms in both the cotton and wheat taxa. Preference for NNA/T codons was heterogeneous among genes with various numbers of introns and was also differential among the exons. SCUB patterns distinguished tetraploid cotton from its diploid progenitor species, as well as bread wheat from its diploid/tetraploid progenitor species, indicating that SCUB in the chloroplast genome partially mirrors the formation of polyploidies.

## Introduction

The single-nucleotide substitution in protein-coding sequences produces either a synonymous codon (SC) or nonsynonymous codon. All but two of the 20 amino acids (methionine and tryptophan) are encoded by at least two synonymous codons (SCs). The situation where one such codon is preferred over others is referred to as “synonymous codon usage bias” (SCUB). SCUB has been proved to reflect the consequence of genetic events such as mutation, genetic drift and natural selection during the evolutionary process [1–4]. Moreover, previous genome-scale analyses indicate that SCUB is heterogeneous in the nuclear genomes of land plants (moss, ferns, gymnosperms to angiosperms) and in organellar genomes from algae to land plants, showing SCUB in both nuclear and organellar genomes is closely associated with plant evolution [5–7].

In polyploid species, which dominate the plant kingdom, genes originating from all of their progenitor species are inherited, but the chloroplast genome is only passed through the maternal gamete. The nuclear genome of a *de novo* formed polyploidy typically experiences perturbation, referred to as “genomic shock” [8]. The phenomenon of genomic shock in the nuclear genome originally described large scale chromosomal rearrangements, but this definition has since been widened to encompass the formation of indels, single nucleotide polymorphisms, and alterations to the epigenome. The nucleotide substitution produces the difference in SCUB of nuclear genes of polyploidies from their ancestors [9]. Over time, most of the genes carried by the chloroplast were either eliminated or transferred into the host’s nuclear genome, which is along with the events of sequence insertions and deletions (indels) that lead to genomic shock [10] and induce local single-nucleotide substitution and other genetic variations [11]. Thus, the interesting question is whether SCUB is affected during the formation of polyploidies.

Intron gain/loss, largely driven by recombination and indel formation, is a key force of the evolution of genomes [12, 13]. SCUB has been proved to associate with intron in the nuclear genome [14]. The frequencies of SCUB are heterogeneous among genes possessing different number of introns, and in genes with the same introns, SCUB frequencies are also different among exons [5]. Moreover, the association between SCUB and intron has been found be affected by the evolution events [5]. Although the effect of intron on SCUB was found to be weak in the chloroplast genome, the evidence for heterogeneity within exon sequence is fragmentary [9]. Thus, whether introns have an effect on the SCUB during the formation of polyploidies is also worthy of been addressed.

The purpose of the present research was to establish whether SCUB in the chloroplast genomes is associated with polyloidization using hexaploid wheat / tetraploid cotton and their tetraploid and diploid progenitors. We found that SCUB in the chloroplast genomes mirrors the formation route of polyploidies.

## Materials and methods

### Chloroplast genome sequences and gene structure

Two contrasting taxa were studied, one the dicotyledonous species belonging to the genus *Gossypium*, as represented by the tetraploid form *G*. *hirsutum* (cotton, AADD) and its two diploid progenitors *G*. *herbaceum* (AA) and *G*. *raimondii* (DD). The other included the hexaploid bread wheat (*Triticum aestivum*, AABBDD), its wild tetraploid progenitor *T*. *dicoccoides* (AABB) and domesticated tetraploid progenitor *T*. *turgidum* (AABB), and its tetraploid progenitors *T*. *urartu* (AA) and *Aegilops tauschii* (DD). Given wheat B subgenome progenitor is unknown, its closely related species *A*. *speltoides* (SS genome) was used as B subgenome progenitor. Besides, the reproductive isolation species *T*. *boeoticum* (AA) and domesticated species *T*. *monococcum* (AA) of *T*. *urartu* were also analyzed.

The chloroplast genome sequences of *G*. *herbaceum* (NC_023215), *G*. *raimondii* (NC_016668) and *G*. *hirsutum* (NC_007944), and of *T*. *urartu* (NC_021762), *T*. *boeoticum* (KC912692), *T*. *monococcum* (NC_021760), *Ae*. *speltoides* (NC_022135), *Ae*. *tauschii* (NC_022133), *T*. *dicoccoides* (KJ614401), *T*. *turgidum* (NC_024814) and *T*. *aestivum* (NC_002762) were all downloaded from GenBank (www.ncbi.nlm.nih.gov/genome/browse#!/organelles/). The intron/exon structure of their protein-encoding genes was derived according to the annotations of chloroplast genomes in the NCBI database. Coding sequences of length which were a multiple of three were deemed to be genes. First codon trinucleotides other than the canonical ATG were deemed to be atypical start codons; in addition to TAA, TAG and TGA, alternative forms of the final three nucleotides were assumed to represent atypical stop codons. The amounts of the protein-coding codons, typical start codon and typical stop conds were listed in S1 Table in S1 File. Codons interrupted by an intron between the first and the second nucleotide were treated as belonging to the downstream exon, while those interrupted between the second and the third nucleotides were deemed to belong to the upstream exon [8].

## Calculation of SCUB indices

The CDS sequences of all protein coding genes in the chloroplast genome of a species were combined into one FASTA sequence, which was used to calculate relative synonymous codon usage (RSCU), codon adaptation index (CAI) and other indices of SCUB with the CodonW 1.4.2 software (https://sourceforge.net/projects/codonw/).

### Calculation of SCUB frequency

Besides RSCU, CAI and other indices of SCUB, we also calculated SCUB frequencies as described previously [8] to compare the difference among species. Briefly, the set of 59 synonymous codons (SCs), encoding 18 of the amino acids was used for the calculations; the five codons including the three stop codons TAA, TAG and TGA, ATG (methionine) and TGG (tryptophan) were excluded. The number of all codons in CDS was calculated by the number of all codons except for TGG, the start and stop codons; atypical start codons (the first three nucleotides are not ATG) and atypical stop codons (the last three nucleotides are not TAA, TAG and TGA) that are rarely present in a few chloroplast genes of some species [15–17]. Total SCUB frequencies were calculated using the ratio of the number of all SCs having A, T, C or G at the third position (abbreviated as NNA, NNT, NNC or NNG) to the number of all codons represented in the coding sequences. The SC frequency for an amino acid encoding by SCs was calculated as the ratio between the number of its SCs ending with C and/or G (NNCs and/or NNGs) and the number of its NNAs or NNTs except for TGG, the start and stop codons, atypical start and atypical stop codons.

RNA editing converting specific cytosine to uridine (C to U) or *vice versa* occurs in some chloroplast transcripts [18–20]. Such conversions in coding sequence affected all three nucleotide positions; however, since RNA editing only rarely converted an SC into a non SC (or *vice versa*), the effect of RNA editing on SCUB frequency was considered to be negligible. Moreover, the C-to-U and U-to-C RNA editing is not annotated in the chloroplast genome database of most species (http://www.ncbi.nlm.nih.gov/genomes/GenomesGroup.cgi?taxid=33090&opt=organelle). Thus, following analyses of SCUB in plastid DNA, the impact of RNA editing was ignored here.

DNA methylation is a major source of DNA variation in the nuclear genome, given that methylated cytosine (5^m^C) is readily converted into thymine [21]. Methylation is mainly present in C of CpG, the conversion of 5^m^C produces TpG in sense strand and CpA in antisense strand. Given the lower selection pressure on the third position of codons, the conversion of NCG to NCA (the second-third position) as well as NC|G to NT|G (the third-next codon’s first position) would be dominant, which leads to the bias to A- and T-ending codons [6]. Thus, the ratios of NXG/NXC (X = A, T, C, or G) can reflect the effect of the second nucleotide on the conversion from G and C to A and T at the third positon, and the ratios of NG|X/NC|X (X = A, T, C, or G) can reflect of first nucleotide of the next codon on the conversion from G and C to A and T at the third position. Based on this, the difference between the ratios of NCG/NCC and NAG/NAC, NGG/NGC, NTG/NTC as well as the difference between the ratios of NC|G/NG|G and NC|A/NG|A, NC|C/NG|C and NC|T/NG|T were calculated to assess the potential association between DNA methylation and SCUB.

### Cluster analysis and Principal Component Analysis (PCA)

Cluster analysis using the SC frequencies and RSCU values of 59 SCs was conducted with the average linkage method and distance measurement of correlation in Minitab 17 statistical software. The dendrogram was generated on the basis of similarity. The SCUB frequencies and RSCU values of 59 SCs were also subjected to principal component analysis in JMP 13 software with default parameters. The factor score coefficients given by the first three PCs were used to generate the scatter plot diagrams.

### Statistical analysis

SCUB frequencies of 18 amino acids were compared to the value 1 to assess the bias to A- and T-ending codons using the one-sample *t* test. The chi square (χ^2^) test was performed to establish the significance of differences in the SCUB frequency between NNAs/Ts and NNCs/Gs, and the amounts of A- and T-ending codons (NNAs/Ts) and C- and G-ending codons (NNCs/Gs) were used for statistical analysis. The significance of differences in SCUB frequency related to the third nucleotide position concerning DNA methylation was analyzed with the χ^2^ test of the cross-table analysis. For example, the difference between NCG/NCC ratio (the second-third nucleotide combination) and NXG/NXC ratio (X = A, G or T respectively) was analyzed by the amounts of NCG, NCC, NXG and NXC; the difference between NC|G/NG|G ratio (the third nucleotide and the first nucleotide of next codon combination) and NC|X/NG|X (X: A, C, or T respectively) was analyzed by the amounts of NC|G, NG|G, NC|X and NG|X. The difference between NXC and NXG SCs of an amino acid encoding by G- and C-ending SCs (Ala, Pro, Ser, Thr, Arg, Gly, Leu and Val) was calculated with the χ^2^ test, and the amounts of NXC and NXG were used for calculation. The difference between the ratios of NCG/NCA of Ala, Pro, Ser, and Thr and the ratios of N(G/T)G/N(G/T)A of Arg, Gly, Leu or Val was calculated with the *t*-test, and the ratios were used for analysis. The difference in the SCUB frequencies between genes with different introns as well as between exons was calculated via the two-sample *t*-test, where the ratios of NNCs/Gs to NNA/Ts in genes with different introns as well as the ratios of NNCs/Gs to NNA/Ts in different exons were used for comparison. The difference in SCUB frequency among genes with different introns as well as among exons in *Gossypium* spp. and *Triticum/Aegilops* spp. was calculated with the two-sample *t*-test, and the ratios of NNC/Gs to NNA/Ts were used for analysis. The difference between the NNCs/Gs to NNAs/Ts ratio and the C/G to A/T ratio in the gene body and whole genome sequences was tested using the χ^2^ test of the cross-table analysis, and the amounts of NNCs/Gs, NNAs/Ts, C/G, A/T were used for comparison. The consistency of SCUB frequency was detected via reliability analysis (model was set as alpha), and Cronbach's Alpha value. The fluctuation was assessed by the coefficient of variation (CV), which is calculated as the ratio of standard deviation to mean. The correlation of SCUB frequencies of 18 amino acids between two species were analyzed with the Pearson’s correlation analysis. P value less than 0.05 was considered significant difference. The statistical analysis was performed with SPSS 19 software.

## Results

### Gene content and structure in the chloroplast genomes

The range in the number of protein-encoding genes harbored by the chloroplast genomes of the three *Gossypium* spp. was 83–86; among the *Triticum/Aegilops* spp., the range was 77–83, except in *T*. *urartu* where only 60 genes were present (Table 1). Most of these genes were free of introns. In the *Gossypium* spp. chloroplast genomes, 11 genes harbored one intron and four harbored two introns. The chloroplast genomes in the diploid and tetraploid *Triticum/Aegilops* spp. included 5–8 genes with one intron and just one gene with two introns. The bread wheat chloroplast genome harbored 11 genes with one intron and two with two introns, and the amount of intron-containing genes was similar to *Gossypium* spp.

**Table 1:** Variation in the number of introns within genes in the chloroplast genomes.

Plants	Species	Genome	Accession	Genes with intros			
				**0**	**1**	**2**	**Total**
**Cotton**	*G*. *herbaceum*	AA	NC_023215	71	11	4	86
	*G*. *raimondii*	DD	NC_016668	68	11	4	83
	*G*. *hirsutum*	AADD	NC_007944	68	11	4	83
**Wheat**	*T*. *urartu*	AA	NC_021762	52	7	1	60
	*T*. *boeoticum*	AA	KC912692	69	7	1	77
	*T*. *monococcum*	AA	NC_021760	72	6	1	79
	*A*. *speltoides*	SS (BB)	NC_022135	71	5	1	77
	*A*. *tauschii*	DD	NC_022133	71	8	1	80
	*T*. *dicoccoides*	AABB	KJ614401	75	6	1	82
	*T*. *turgidum*	AABB	NC_024814	75	6	1	82
	*T*. *aestivum*	AABBDD	NC_002762	70	11	2	83

### Start codons, stop codons and internal stop codons in the chloroplast genome

Besides ATG and three typical stop codons, atypical start (not ATG) and stop (not TAA, TGA and TAG) codons are present in some chloroplast genes [15–17]. The most commonly used transcription initiation codon was ATG (Table 2). ACG was the sole non-standard start codon represented in the *Gossypium* spp. chloroplast genomes, while the *Triticum/Aegilops* spp. featured a more diverse range of non-standard codons: the A genome wild diploids *T*. *urartu* and *T*. *boeoticum*, the surrogate B genome diploid *Ae*. *speltoides*, the D genome diploid *Ae*. *tauschii* and bread wheat used ACG, but this was not the case for either the A genome domesticate *T*. *monococcum* or the two AB tetraploids *T*. *dicoccoides* and *T*. *turgidum*. GTG is a common atypical start codon present in the chloroplast genomes of land plants [6]. Examples of the use of GTG arose in *T*. *urartu*, *T*. *dicoccoides*, *T*. *turgidum* and *T*. *aestivum*, but not in either *T*. *boeoticum* or *T*. *monococcum*; GTG was not found as start codon in the *Gossypium* spp. Occurrences of both ATC and CTG were identified in both tetraploid accessions of wheat.

**Table 2:** Codon usage for transcription initiation and termination used by genes in the chloroplast genomes.

Plant	Species	Start codon					Stop codon			Internal stop codon
		ATG	ACG	GTG	ATC	CTG	TAA	TAG	TGA	TAA
**cotton**	*G*. *herbaceum*	85	1				50	19	17	
	*G*. *raimondii*	82	1				47	19	17	
	*G*. *hirsutum*	82	1				47	19	17	
**wheat**	*T*. *urartu*	57	1	1			35	12	12	
	*T*. *boeoticum*	75	1				40	20	16	
	*T*. *monococcum*	78					43	19	16	
	*A*. *speltoides*	76	1				40	19	18	
	*A*. *tauschii*	78	1				43	19	17	
	*T*. *dicoccoides*	78		2	1	1	45	20	17	
	*T*. *turgidum*	78		2	1	1	45	20	17	
	*T*. *aestivum*	79	2	2			47	19	17	2

Among three typical stop codon, the most frequently used stop codon was TAA, with TAG and TGA occurring at a similar, lesser frequency (Table 2). Atypical stop codons were present in the chloroplast genomes of some species [6], but there was no atypical stop codon in both cotton and wheat species (Table 2). Internal stop codons rarely exist in gene body of the organellar genomes [16, 22], and they are eliminated via the uridine-to-cytidine (U-to-C) editing that is a kind of RNA processing for a few organellar genes in some species [23, 24]. Here, internal stop codons were only identified in bread wheat (Table 2).

### SCUB patterns among polyploids and progenitor species

The values of relative synonymous codon usage (RSCU) of 59 SCs ranged from 0.35 (AGC in *G*. *hirsutum*) to 1.83 (TTA) in the *Gossypium* spp., and were respectively similar among diploid and tetraploid species (coefficients of variation (CV) were 0 ~ 0.024) (S2 Table in S1 File). RSCU values were 0.30 (CTG in *T*. *aestivum*) to 2.06 (TTA in tetraploid and hexaploid species) in the *Triticum/Aegilops* spp., and RSCU values of some codons fluctuated (CV = 0.004 ~ 0.049), among which AGC and GGC had higher and lower RSCU respectively in tetraploid and hexaploid species, while TTA had lower RSCU in *T*. *urartu*. Codon adaption index (CAI) and other indices were similar among *Gossypium* spp. and *Triticum/Aegilops* spp. (S3 Table in S1 File).

**Fig 1:**
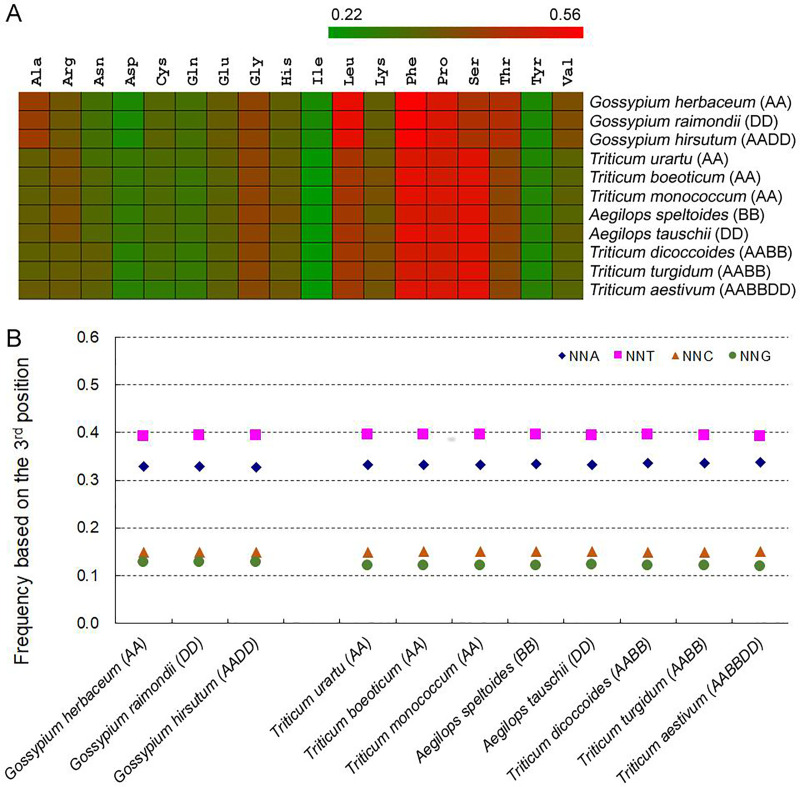
SCUB frequency in genes harboured by the chloroplast genomes. (A) The ratio between the number of NNC/NNG SCs and NNA/NNT SCs of 18 amino acids (Met and Trp not included). NNCs/Gs: the number of synonymous codons (SCs) as C or G as their final bases; NNAs/Ts: the number of SCs as A or T as their final base. N denotes any base. (B) The frequency of NNA, NNT, NNC and NNG codons. The frequency is defined as the ratio between the number of all SCs with A, T, C or G as the final base and the full set of encoding codons. The statistical comparison was conducted with chi square (χ^2^) test using the amounts of codons as showed in S2 Table in S1 File.

Among 61 codons encoding 20 amino acids, A- and T-ending codons (NNAs and NNTs) were more frequent than C- and G-ending codons (NNCs and NNGs) in chloroplast genes (S1 Fig in S1 File). The pattern of SCUB frequencies of 59 SCs were almost consistent to that of RSCU values (S2 Fig in S1 File). To gain a direct view of SCUB, we defined SCUB frequency of a given amino acid encoded by synonymous codons (SCs) as the ratio of NNCs and NNGs (NNCs/Gs) number to NNAs/Ts number (Fig 1A). The SCUB frequencies of the individual amino acids ranged from 0.246 (Leu in *G*. *raimondii*) to 0.552 (Pro in *G*. *herbaceum*) among the *Gossypium* spp. and from 0.216 (Leu in *T*. *monococcum*) to 0.522 (Pro in *Ae*. *tauschii*) among the *Triticum/Aegilops* spp. (Fig 1A). The coefficients of variation associated with these values were, respectively, 0.24 and 0.22 (S4 Table in S1 File). The mean SCUB frequency across all of the amino acids was 0.378 for the *Gossypium* spp. genes and 0.367 for the *Triticum/Aegilops* spp. genes, significantly lower than 1 (P < 1.00×10^−15^, one-sample *t*-test).

The SCUB frequency of each amino acid was comparable among either cotton or wheat accessions (Fig 1A). The similarity of usage across the three *Gossypium* spp., as measured by the correlation coefficient *r* was ~0.999, and varied from 0.981 to 0.9999 across the *Triticum/Aegilops* spp. (S5 Table in S1 File). The *r* values derived from comparisons between diploids were a little higher than those derived from comparisons between diploids and polyploids: for instance, the *r* values for the comparisons *T*. *urartu vs Ae*. *speltoides*, *T*. *urartu vs T*. *dicoccoides* and *T*. *urartu vs* hexaploid *T*. *aestivum* were, respectively 0.994, 0.982 and 0.981. However, *r* values within diploid accessions were not higher than those within tetraploid accessions of wheat.

There was some variation in the SCUB frequency between individual amino acids (Fig 1A). For example, among the *Gossypium* spp., leucine was associated with the second largest SCUB frequency (~0.52), but among the *Triticum/Aegilops* spp., its frequency was only intermediate (~0.43); meanwhile, serine was associated with an intermediate SCUB frequency (~0.45) among the *Gossypium* spp., but with a large one (~0.53) among the *Triticum/Aegilops* spp. Cronbach’s alpha coefficient, used to assess the consistency of SCUB frequencies, was 1.000 among the *Gossypium* spp. and 0.999 among the *Triticum/Aegilops* spp., but fell to 0.947 in comparisons between the *Gossypium* spp. and the *Triticum/Aegilops* spp.

The ratio between NNC/G and NNA/T trinucleotides was lower than that between GC and AT dinucleotides in both gene body and the genomic sequence (S6 Table in S1 File). SCUB in the chloroplast genes was directly represented by the total SCUB frequencies of NNA, NNT, NNC and NNG, which were respectively defined as the ratios of the numbers of all NNAs, NNTs, NNCs and NNGs to the codon number of all CDS in a chloroplast genome. NNA/T codons were more abundant than NNC/G codons (Fig 1B; P < 0.001, χ^2^ test) (S4 Table in S1 File). NNT frequency was obviously higher than NNA frequency, while NNC frequency was quite close to NNG frequency, especially in cotton spp. Within the genus *Gossypium*, the frequency of the four codons was well conserved (CV = 0.010~0.020), as was also the case within the *Triticum/Aegilops* complex (CV = 0.003~0.007). However, there was a significant difference in the frequencies of NNA and NNG codons between the two groups of species (P = 0.003 and 2.0×10^−7^, *t*-test using the frequencies of NNA or NNG respectively), with the *Triticum/Aegilops* spp. showing a higher frequency of NNA and a lower one of NNG codons. The frequencies of NNT and NNG were comparable between the two groups of species (P = 0.153 and 0.557, *t*-test).

### The effect on SCUB of intron number

**Fig 2:**
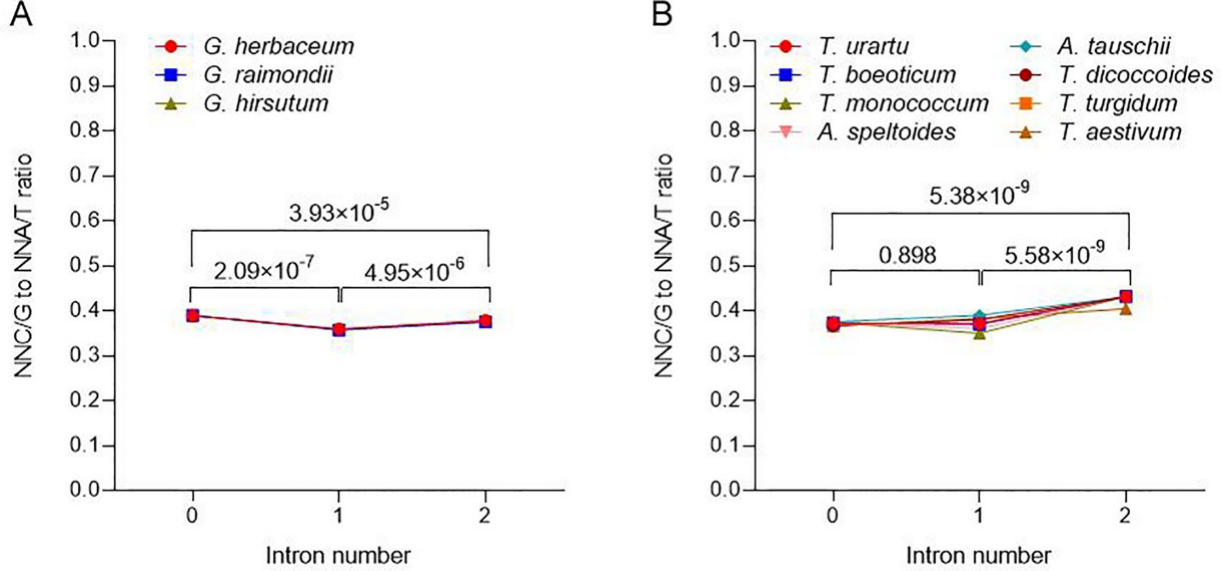
The influence of intron number on SCUB. (A) *Gossypium* spp., (B) *Triticum/Aegilops* spp. NNC/G to NNA/T ratio was defined as the ratio between the number of all SCs with C or G as the final base to the number of all SCs as A or T as the final base. N denotes any base. The statistical significance of differences among the sets of genes harboring a given number of introns were calculated with two-sample *t*-test (P < 0.05 was considered to be significantly different), and NNC/G to NNA/T ratios were used for analysis.

SCUB is differential in genes possessing various introns in nuclear genomes [5], and the heterogeneity in organellar genomes also mirrors the evolution of plants [6]. To assess whether SCUB frequency was influenced by the number of introns present, the ratio between the number of NNC/G codons and the number of NNA/T codons was compared for genes varying with respect to their intron number (Fig 2). Among the *Gossypium* spp., the ratios of genes without or with introns exhibited considerable similarity (CV = 0.0005 (*G*. *herbaceum*), 0.0025 (*G*. *raimondii*) and 0.0049 (*G*. *hirsutum*)) (Fig 2A). However, there was a significant difference among gene with no, one and two introns (P < 4.0×10^−5^, two-sample *t*-test). The genes bearing just one intron exhibited the lowest SCUB frequency, and those lacking any intron the highest. Among the *Triticum/Aegilops* spp., the set of intron-less genes exhibited almost the same SCUB frequency, whereas the frequency was more variable when introns were present (CV = 0.050~0.109) (Fig 2B): genes harboring either zero or one intron exhibited a similar SCUB frequency (P = 0.898, two-sample *t*-test), which was significantly lower in genes harboring two introns (P = 5.38×10^−9^ and 5.58×10^−9^, two-sample *t*-test). The SCUB frequency in genes harboring one intron varied among the *Triticum/Aegilops* spp., while those harbouring two introns exhibited a lower SCUB frequency in bread wheat than in any of the diploid or tetraploid accessions.

### The influence of exon location on SCUB frequency

**Fig 3:**
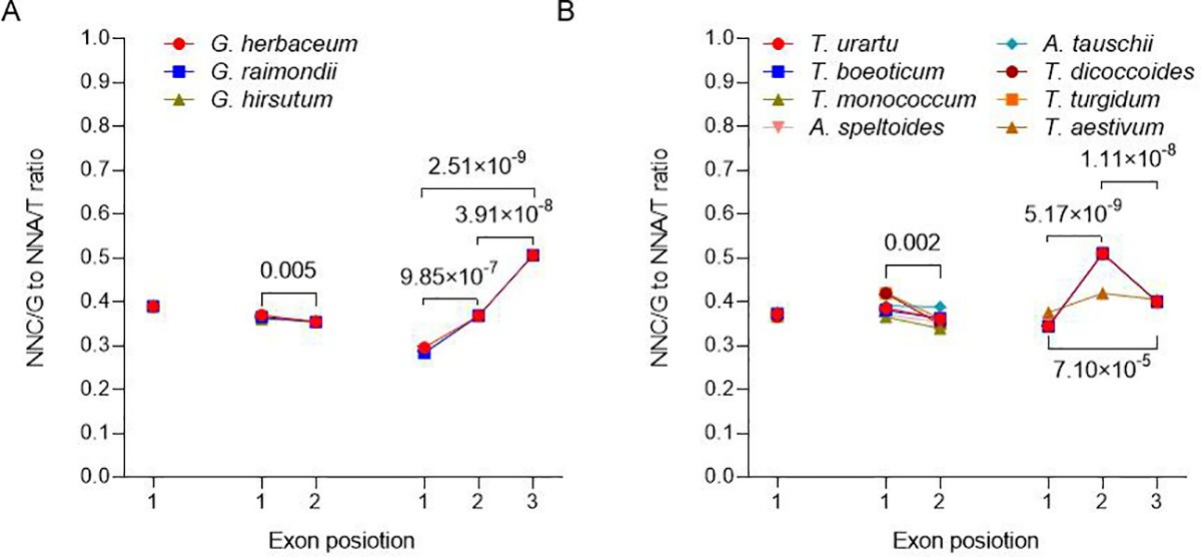
The influence of exon position on SCUB. (A) *Gossypium* spp., (B) *Triticum/Aegilops* spp. NNC/G to NNA/T ratio was defined as the ratio between the number of all SCs with C or G as the final base to the number of all SCs as A or T as the final base. N denotes any base. The statistical significance of differences among exons were calculated with two-sample *t*-test (P < 0.05 was considered to be significantly different), and NNC/G to NNA/T ratios were used for analysis.

SCUB frequency is different among exons in nuclear and organellar genes [5, 6], so we further analysed whether this rule is present in the chloroplast genomes of polyploidies using the NNC/G to NNA/T ratio. The SCUB frequency in exon sequence among the *Gossypium* spp. was not greatly affected by the presence of introns (CV = 0~0.025) (Fig 3). However, in genes where two exons were present, the frequency was significantly lower in the second exon than in the first (P = 0.005, two-sample *t*-test) (Fig 3A). In the genes which featured three exons, the frequency was lowest in the first exon and highest in the third exon. The situation in the *Triticum/Aegilops* spp. chloroplast genome was somewhat different (Fig 3B). Here, the SCUB frequency in intronless genes (just as in the *Gossypium* spp. chloroplast genome) was comparable across the taxa (CV = 0.010). In coding sequences separated into two exons, the frequency in the first exon was higher than the second exon (P = 0.002, two-sample *t*-test); the frequency of the first exon was higher in both the hexaploid and tetraploid accessions than in the diploids (P = 6.32×10^−4^, two-sample *t*-test), but the frequency of the second exon was comparable among the taxa. In genes split into three exons, the lowest SCUB frequency occurred within the first exon and the highest within the second exon (P < 7.10×10^−5^, two-sample *t*-test). The frequency in each of three exons of three-exon genes was similar in the diploid and tetraploid accessions of wheat. In hexaploid wheat, the frequency in the first exon was higher than in the lower ploidy species (P = 2.29×10^−91^, one-sample *t*-test), the second’s frequency was lower (P = 1.02×10^−91^, one-sample *t*-test) and the third’s was not different, so that the difference of SCUB frequencies among three exons (CV = 0.056) were weaker than that of ancestors (CV = 0.203).

### A possible association between DNA methylation and SCUB

**Fig 4:**
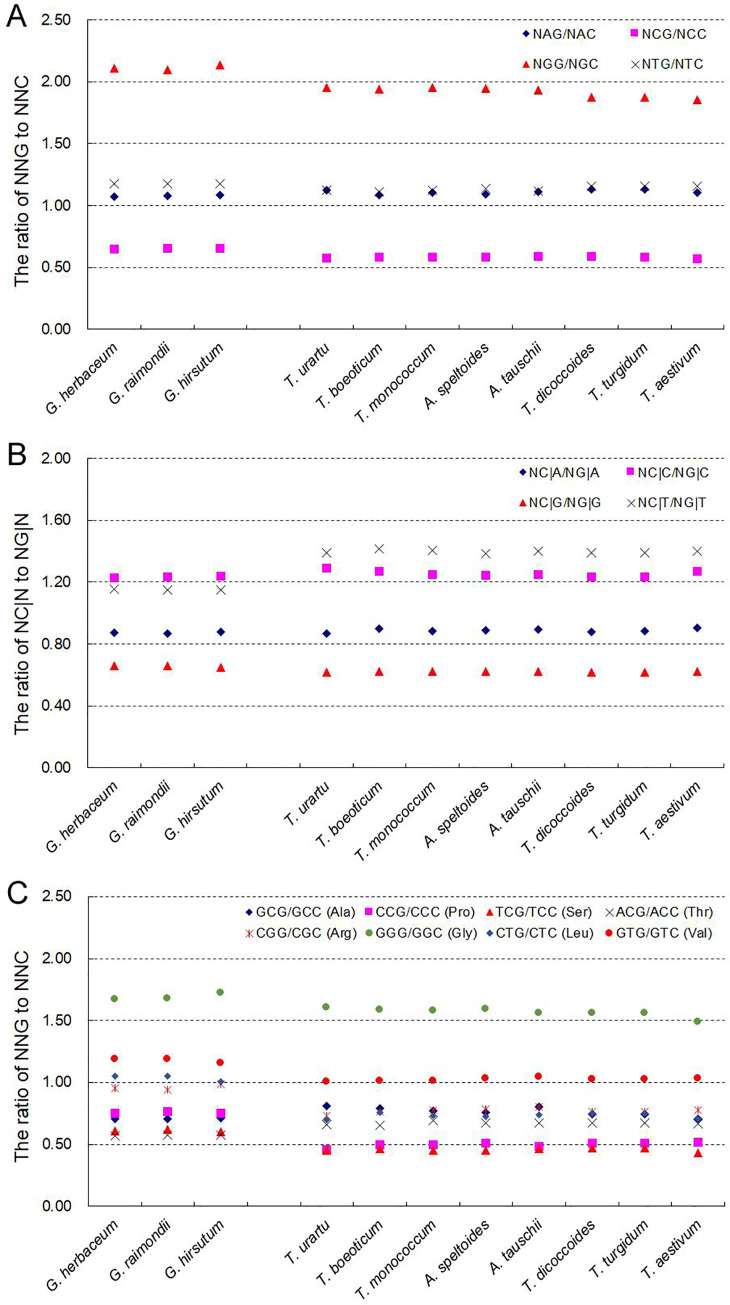
The association between SCUB frequency and DNA methylation-induced conversion of cytosine to thymine. (A) The ratio between the numbers of NNG codons to NNC codons, according to the identity of the second position nucleotide. (B) The ratio of the number of NC|N adjacent codons to NG|N adjacent codons, according to the identity of the first position nucleotide in the downstream codon. (C) The ratio of the number of NNG to NNC codons encoding a given amino acid. The statistical comparison was conducted with chi square (χ^2^) test using the amounts of codons as shown in S7, S8 and S10 Tables in S1 File; in panel C, the difference of the ratios between Ala, Pro, Ser, Thr and Arg, Gly, Leu and Val was calculated with two-sample *t*-test (P < 0.05 was considered to be significantly different) as shown in S9 Table in S1 File.

CpG methylation is a driver of bias to A- and T-ending SCs in the nuclear genome of land plants [5]. To investigate this possible association in chloroplast genes of polyploidies, we attempted to determine the influence on SC frequency of the identity of the nucleotide in the second position of NNC or NNG codons and that of the nucleotide in the first position of the downstream codon. In both the *Gossypium* spp. and the *Triticum/Aegilops* spp. genes, the NCG / NCC, NGG / NGC, NAG / NAC and NTG / NTC ratios all differed significantly (P = 9.56×10^−60^ ~ 1.12×10^−42^, χ^2^ test). The NCG / NCC ratios were significantly lower than 1 (P = 3.07×10^−47^ ~ 3.21×10^−45^ in the *Gossypium* spp., 8.61×10^−29^ ~ 2.25×10^−26^ in the *Triticum/Aegilops* spp., χ^2^ test), but NGG / NGC, NAG / NAC and NTG / NTC ratios were higher than 1 (S7 Table in S1 File). In particular, the ratio of NCG / NCA indicating the methylation-mediated conversion was significantly lower than the ratios of NGG / NGC, NAG / NAC and NTG / NTC (P = 2.722×10^−13^ ~ 5.758×10^−13^, χ^2^ test) (Fig 4A; S8 Table in S1 File). This indicated that in comparison with A, G and T at the second position, C at the second position had a stronger effect on decreasing the bias of G at the third position, suggestive of potential association between methylation-mediated conversion and SCUB.

Similarly, drastic difference was found in the ratios of NC|G / NG|G, NC|A / NG|A, NC|T / NG|T and NC|C / NG|C (P = 6.04×10^−32^ ~ 9.35×10^−25^, χ^2^ test). The NC|G / NG|G ratios were obviously lower than 1 (P = 2.16×10^−25^ ~ 7.48×10^−24^ in the *Gossypium* spp., 2.06×10^−23^ ~ 8.91×10^−21^ in the *Triticum/Aegilops* spp., χ^2^ test), but NC|T / NG|T and NC|C / NG|C ratios were higher than 1; NC|A / NG|A ratios were also lower than 1, but they were higher than NC|G / NG|G ratios, and the difference from 1 (P = 3.98×10^−4^ ~ 0.035, χ^2^ test) was not as drastic as NC|G / NG|G ratios (S7 Table in S1 File). The ratio of NC|G / NG|G indicating the methylation-mediated conversion was significantly lower than the ratios of NC|A / NG|A, NC|T / NG|T and NC|C / NG|C (P = 3.251×10^−6^ ~ 2.878×10^−14^, χ^2^ test) (Fig 4B; S8 Table in S1 File), which was also consistent with the occurrence of methylation-mediated conversion. At the same time, there was no discernible difference between the polyploid and diploid accession in either the *Gossypium* spp. or the *Triticum/Aegilops* spp. for any of the ratios (the relevant Cronbach alpha coefficients were, respectively, 0.9998 and 0.9999, and 0.9996 and 0.9995) (Fig 4A and 4B).

A sample of C- and G-ending SC pairs of amino acids sharing the same nucleotides in their first and second positions was analyzed to identify any effect of the second nucleotide on the frequency of these SCs (Fig 4C). For the *Gossypium* spp. genes, the NCG / NCC (encoding alanine, proline, serine and threonine) ratios varied from 0.568 to 0.765, and NCG had significantly higher frequencies than NCC (P = 0.011 ~ 2.50×10^−8^, χ^2^ test) (S9 and S10 Tables in S1 File). Oppositely, among N(G/T)G / N(G/T)C (encoding arginine, glycine, leucine and valine) ratios (0.944 to 1.723), NGG/NGC ratios of glycine were larger than 1 (P = 1.84×10^−9^ ~ 8.64×10^−9^, χ^2^ test), N(G/T)G / N(G/T)C ratios of other three amino acids were around 1 (P = 0.093 ~ 0.959, χ^2^ test). The NCG / NCC ratios were significantly higher than N(G/T)G / N(G/T)C ratios (P = 0.007 ~ 0.010, two-sample *t*-test) (S9 Table in S1 File). Similarly, for the *Triticum/Aegilops* spp. genes, the NCG / NCC ratios (0.433 ~ 0.808), and the frequencies of NCG were significantly higher than those of NCC (P = 0.042 ~ 1.67×10^−14^, except for Ala in *T*. *Urartu* (0.074) and *A*. *tauschii* (0.060), χ^2^ test) (S9 and S10 Tables in S1 File). Among N(G/T)G / N(G/T)C ratios (0.697 to 1.608), NGG/NGC ratios of glycine were larger than 1 (P = 3.82×10^−6^ ~ 4.71×10^−5^, χ^2^ test), NTG/NTC ratios of valine were near to 1 (P = 0.722 ~ 0.950, χ^2^ test), but NGG/NGC (arginine) and NTG/NTC (leucine) ratios were lower than 1 (P = 0.006 ~ 0.140, χ^2^ test). The NCG / NCC ratios were also significantly higher than N(G/T)G / N(G/T)C ratios (P = 0.033 ~ 0.045 with the exception of *T*. *urartu* P = 0.059, two-sample *t*-test) (S9 Table in S1 File). Among the *Triticum/Aegilops* spp. genes, the NCG / NCC ratios of serine and proline were significantly smaller than the other ratios, followed by the NCG / NCC ratios of threonine and alanine that were similar to the NG/TG / NG/TC (arginine and leucine) ratios. Among both the *Gossypium* spp. and *Triticum/Aegilops* spp. genes, the NNG / NNC ratios of eight amino acids did not differ between the polyploid and diploid species (Cronbach alpha coefficients of, respectively, 0.9991 and 0.9995).

### SCUB in the chloroplast genome mirrors the effect of polyploidization

**Fig 5:**
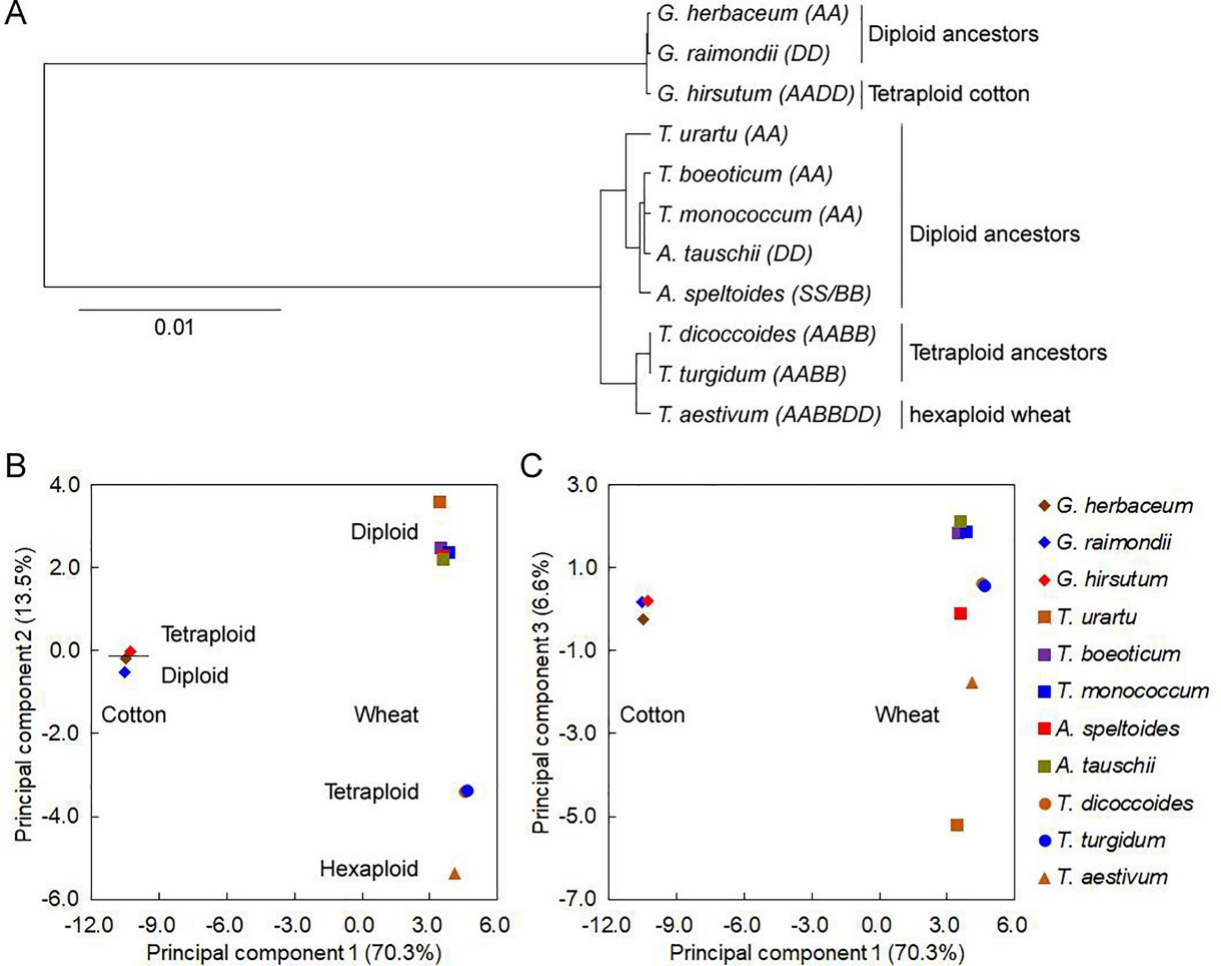
A cluster and principal component analysis of SCUB frequency. (A) A clstuer tree based on SCUB frequency at the set of 59 SCs. (B, C) A two dimensional representation of the PCA based on (B) PC1 and PC2, (C) PC1 and PC3 score coefficients. SCUB frequency was calculated from the ratio between the number of each SC and the number of total SCs.

A cluster based on SCUB frequencies at the set of 59 codons is shown in Fig 5A and S3A and S3B Fig in S1 File. The *Gossypium* spp. and *Triticum/Aegilops* spp. formed two distinct clades. In the former clade, the diploids presented as a sub-clade, differentiated from the tetraploid; in the latter, the diploids also formed a sub-clade distinct from the polyploids, which in turn were differentiated from one another on the basis of ploidy level. The cladistic analysis was supported by the outcome of the PCA (Fig 5B and 5C; S3C-S3E Fig in S1 File). The first principal component (PC1) distinguished the *Gossypium* spp. from the *Triticum/Aegilops* spp.; PC2 separated tetraploid *G*. *hirsutum* from the two diploid *Gossypium* spp., and bread wheat from the tetraploid *Triticum* and diploid *Aegilops* and *Triticum* species (Fig 5B). The PC2 factor score coefficient (FSC) associated with tetraploid *G*. *hirsutum* was larger than that of either of the two diploids. In contrast, the hexaploid bread wheat PC2 FSCs were rather lower than the tetraploid’s and diploids’, and FSCs of diploid accessions were the highest. Along PC3, diploid ancestors were scattered from each other (Fig 5C). *T*. *urartu* was associated with the smallest FSC and was well separated from the remaining *Triticum/Aegilops* spp. *T*. *boeoticum* and *T*. *monococcum*, which like *T*. *urartu*, are both A genome diploids, appeared to be more closely related to the D genome ancestor *Ae*. *tauschii*, and were associated with a large FSC. Hexaploid bread wheat closed to *T*. *urartu* than other species, while tetraploid accessions clustered with *T*. *boeoticum* and *T*. *monococcum*. *Ae*. speltoides, chosen to represent the B genome donor, mapped to a position intermediate between the tetraploids and the bread wheat. Similar results were found when RSCU values were used for the cluster analysis and PCA (S4 Fig in S1 File), except that the distribution of the *Gossypium* spp. and the *Triticum/Aegilops* spp. on PC3 axis (S4C Fig in S1 File). These findings indicate the complication of SCUB in plastid genes of polyploidies.

To clearly outline the complication, individual PCAs for the *Gossypium* spp. and *Triticum/Aegilops* spp. genes were performed (S3C-S3E Fig in S1 File). For the *Gossypium* spp. genes, PC1 distinguished between the tetraploid and the two diploids, with the A genome donor mapping further from the crop species than the D genome donor (S4C Fig in S1 File). For the *Triticum/Aegilops* spp. genes, PC1 separated the various ploidy levels from one another. *T*. *urartu* and other diploid progenitor species were separated in both PC1 and PC2 axis, showing the specificity of A genome ancestor (S3D Fig in S1 File). Along PC3, tetraploid and bread wheat were separated, but the FSCs of the diploid progenitor species were close to 0, meaning that this PC defined a difference between tetraploid and bread wheat (S3E Fig in S1 File). Together with the data of phylogenic tree and PCA, it was demonstrates that SCUB can reflect the difference of polyploidies and ancestors.

## Discussion

### SCUB in chloroplast genes targets NNA/T codons in polyploids and their progenitors

In land plants, the bias to either NNA/T or NNC/G of SCs is present in the nuclear genomes, but it appears to be limited to NNA/T SCs in the chloroplast genomes [5, 25, 26]. The present analysis, which aimed to establish whether this bias was affected by the ploidy level of the plant, showed that the preference for NNA/T SCs was shared between the polyploid crop species cotton and bread wheat and their various progenitor species (Fig 1; S1 Fig in S1 File). The bias extended beyond coding codons, and was also found in the stop codons and the internal stop codons (Table 2). Genomic shock is a force to induce genetic variation such as nuclear substitution in the nuclear genome [27], and including nuclear substitution mostly result in the bias to A and T [14]. During the rapidly evolution of organellar genomes, recombination and the formation of indels lead to genomic shock, and therefore induces signal nucleotide change [27]. The may be a cause for favoring the retention of NNA/T SCs. Nevertheless, there was no evidence of any difference between the polyploid and diploid forms for this bias (Fig 1), which implies that similar selection pressures operated at the level of SCs during and after the allopolyploidization events.

### SCUB within chloroplast genes differs between polyploids and their progenitors

Genomic rearrangement leads to a strong genomic shock, the force of genomic variation during natural evolution and diploydization of polyploidies, and polyploidization is one of the major drivers of genome evolution [28–31]. A prior analysis of SCUB in the nuclear and organellar genomes has suggested that the phenomenon is somewhat taxon-dependent [5, 6]. Here, although the total frequency of SCUB did not differ markedly between polyploid and progenitor diploid forms (Fig 1), its manifestation in the coding sequence of chloroplast genome of both polyploids was distinct from that present in their progenitor species (Fig 5). This indicates that SCUB of chloroplast genes has altered in polyploidies in comparison with their progenitors.

It has been suggested that SCUB in both the nuclear and chloroplast genomes mirrors the evolution of plants [8, 9]. The present analysis revealed that the difference of SCUB in the chloroplast genome was exhibited in an order from diploid progenitor to polyploid form in both cotton and wheat (Fig 5C), which does confirm that it was responsive to the evolutionary events associated with allopolyploidization. Secondly, the orders of cotton and wheat are opposite along the FSC axis (Fig 5C), implying the diversity of alteration of SCUB in chloroplast genes during the formation of polyploids.

Intriguingly, the SCUB exhibited by genes in the chloroplast genome of *T*. *urartu* (the donor of the bread wheat A genome) differed markedly from what was observed in its close relatives *T*. *boeoticum* and *T*. *monococcum*, in which codon usage resembled that present in both the surrogate B genome donor (*Ae*. *speltoides*) and the D genome donor (*Ae*. *tauschii*) (Fig 5C; S3 Fig in S1 File). Given that SCUB reflects a balance between mutation, genetic drift and natural selection [3, 4], the disconnect between SCUB and the well established phylogenetic relationships between the diploid *Triticum/Aegilops* species suggests that their chloroplast genomes have experienced different selection pressures during the two rounds of allopolyploidization involved in the formation of bread wheat. Consistent with this possibility, the correlation coefficients associated with SCUB frequency were rather variable (S11 Table in S1 File).

### SCUB in polyploid taxa is affected by the presence of introns

As a major event in eukaryotic genomes [32], intron evolution can results in nuclear substitution in exon sequence, which commonly prefers to lower GC content [33]. Previously it has been established that in both the nuclear and organellar genome, the frequency of NNA/T SCs rises as the intron number increases [8]. Consistent with this trend, SCUB was shown here to be influenced by intron number in the chloroplast genomes of both *Gossypium* spp. and *Triticum/Aegilops* spp. (Fig 2). Nuclear genes harboring a higher number of introns are thought to experience a greater selection pressure, the result of which tends to favor the retention of NNA/T codons [34, 35]. In contrast, in the chloroplast, there appears to be a preference for NNC/G codons in genes with more introns (Fig 2). Polyploidization seems to have had a neutral effect on the relationship between intron number and SCUB frequency, although the indication is that the overall SCUB frequency in the bread wheat chloroplast genome was somewhat lower than in that of its progenitor species (Fig 2).

Nucleotide substitution is induced by intron evolution in adjacent exons, because indels have proved to perform an efficient role in nucleotide substitution in several hundred bases [11, 36]. Moreover, interstitial exons have a more bias to of NNA/T SCs than terminal ones [8]. Within the chloroplast genome, the SCUB frequency among exonic sequence was unequal with different patterns in the chloroplast genomes of both the *Gossypium* spp. and the *Triticum/Aegilops* spp. (Fig 3). While SCUB in exonic sequence was unaffected by polyploidization in the *Gossypium* genus, there was considerable heterogeneity among the *Triticum/Aegilops* spp., showing the complicated preference of SCUB in chloroplast genes during polyploidization. It should be noted that the number of genes possessing intron(s) is low (Table 2), which may result in the pattern of SCUB is non-linear in the number of introns (Fig 3), so the effect of intron on SCUB still needs to be accurately confirmed in the future.

### DNA methylation is a possible force for SCUB formation in chloroplast genomes

The conversion of methylated cytosine to thymine is a kind of genetic variation [37], which is a force for the bias towards NNA/T SCs in plant nuclear genomes [5]. The DNA methylation was found to had a potential effect on SCUB in organellar genomes [6], although some reports show that cytosine methylation is not rich in the chloroplast genome [8, 38]. Here, SCUB generated by DNA methylation was inferred in the chloroplast genomes of both the *Gossypium* spp. and the *Triticum/Aegilops* spp. (Fig 4). Given that alterations to the epigenome are commonplace during polyploidization, the differences and similarities observed between the chloroplast and nuclear genome with respect to SCUB driven by DNA methylation likely represents a research topic of continuing interest to evolutionary studies.

## Supporting information

S1 File (DOC)
