# Supplementary material for: Polyploidization is accompanied by synonymous codon usage bias in the chloroplast genomes of both cotton and wheat
Source: PLoS One. 2020 Nov 19;15(11):e0242624. doi: 10.1371/journal.pone.0242624 (PMC7676672; doi:10.1371/journal.pone.0242624)
Supplement: S1 File — (DOC) [file pone.0242624.s001.doc]

**S1 Table. The amounts of codons in the plastid genomes.**

| **Amino** | **Codon** | **Cotton** |  |  | **Wheat** |  |  |  |  |  |  |  |
| --- | --- | --- | --- | --- | --- | --- | --- | --- | --- | --- | --- | --- |
| **acid** |  | ***G. herbaceum*** | ***G. raimondii*** | ***G. hirsutum*** | ***T. urartu*** | ***T. boeoticum*** | ***T. monococcum*** | ***A. speltoides*** | ***A. tauschii*** | ***T. dicoccoides*** | ***T. turgidum*** | ***T. aestivum*** |
| **Ala** | **GCA** | 383 | 378 | 367 | 328 | 342 | 349 | 350 | 348 | 373 | 373 | 379 |
| **Ala** | **GCT** | 628 | 627 | 630 | 486 | 514 | 519 | 523 | 523 | 545 | 545 | 542 |
| **Ala** | **GCC** | 249 | 246 | 248 | 156 | 165 | 168 | 172 | 170 | 180 | 180 | 192 |
| **Ala** | **GCG** | 176 | 173 | 177 | 126 | 130 | 130 | 130 | 137 | 134 | 134 | 136 |
| **Arg** | **AGA** | 463 | 459 | 453 | 295 | 325 | 325 | 331 | 335 | 352 | 352 | 362 |
| **Arg** | **CGA** | 370 | 369 | 370 | 212 | 228 | 234 | 237 | 240 | 244 | 244 | 255 |
| **Arg** | **CGT** | 334 | 330 | 331 | 224 | 243 | 253 | 252 | 256 | 276 | 276 | 278 |
| **Arg** | **AGG** | 183 | 178 | 176 | 114 | 123 | 124 | 126 | 129 | 120 | 120 | 126 |
| **Arg** | **CGC** | 126 | 124 | 124 | 96 | 101 | 100 | 105 | 102 | 106 | 106 | 109 |
| **Arg** | **CGG** | 120 | 117 | 122 | 70 | 77 | 78 | 82 | 82 | 81 | 81 | 85 |
| **Asn** | **AAT** | 973 | 964 | 966 | 516 | 553 | 552 | 561 | 559 | 577 | 577 | 598 |
| **Asn** | **AAC** | 300 | 295 | 297 | 160 | 182 | 180 | 182 | 183 | 201 | 201 | 204 |
| **Asp** | **GAT** | 866 | 865 | 864 | 484 | 516 | 516 | 536 | 526 | 550 | 550 | 559 |
| **Asp** | **GAC** | 220 | 216 | 217 | 142 | 150 | 150 | 152 | 155 | 150 | 150 | 152 |
| **Cys** | **TGT** | 226 | 224 | 222 | 144 | 154 | 154 | 155 | 155 | 163 | 163 | 169 |
| **Cys** | **TGC** | 74 | 74 | 75 | 46 | 49 | 49 | 50 | 50 | 51 | 51 | 48 |
| **Gln** | **CAA** | 719 | 710 | 713 | 438 | 472 | 477 | 481 | 481 | 505 | 505 | 517 |
| **Gln** | **CAG** | 222 | 220 | 220 | 137 | 140 | 142 | 147 | 146 | 146 | 146 | 149 |
| **Glu** | **GAA** | 1022 | 1010 | 1009 | 693 | 714 | 727 | 734 | 731 | 775 | 775 | 788 |
| **Glu** | **GAG** | 360 | 355 | 357 | 240 | 240 | 243 | 253 | 248 | 260 | 260 | 269 |
| **Gly** | **GGA** | 721 | 711 | 714 | 507 | 529 | 530 | 539 | 541 | 570 | 570 | 592 |
| **Gly** | **GGT** | 573 | 571 | 575 | 410 | 440 | 441 | 444 | 450 | 468 | 468 | 486 |
| **Gly** | **GGC** | 197 | 195 | 191 | 143 | 152 | 153 | 156 | 157 | 163 | 163 | 171 |
| **Gly** | **GGG** | 329 | 327 | 329 | 230 | 241 | 242 | 249 | 245 | 254 | 254 | 255 |
| **His** | **CAT** | 476 | 468 | 471 | 304 | 307 | 310 | 310 | 317 | 323 | 323 | 332 |
| **His** | **CAC** | 165 | 162 | 160 | 106 | 108 | 110 | 116 | 112 | 113 | 113 | 120 |
| **Ile** | **ATA** | 693 | 683 | 690 | 431 | 469 | 468 | 483 | 482 | 492 | 491 | 513 |
| **Ile** | **ATT** | 1105 | 1097 | 1099 | 717 | 771 | 775 | 781 | 779 | 815 | 814 | 829 |
| **Ile** | **ATC** | 449 | 448 | 440 | 256 | 275 | 269 | 276 | 279 | 291 | 292 | 292 |
| **Leu** | **CTA** | 401 | 392 | 395 | 270 | 281 | 283 | 290 | 293 | 304 | 304 | 316 |
| **Leu** | **CTT** | 576 | 575 | 572 | 403 | 418 | 424 | 431 | 430 | 448 | 448 | 455 |
| **Leu** | **TTA** | 848 | 838 | 840 | 618 | 673 | 683 | 690 | 692 | 725 | 725 | 742 |
| **Leu** | **CTC** | 190 | 184 | 187 | 132 | 136 | 141 | 141 | 144 | 145 | 145 | 151 |
| **Leu** | **CTG** | 200 | 194 | 188 | 92 | 103 | 103 | 102 | 106 | 109 | 109 | 107 |
| **Leu** | **TTG** | 571 | 570 | 568 | 348 | 356 | 364 | 366 | 368 | 382 | 382 | 389 |
| **Lys** | **AAA** | 1045 | 1026 | 1023 | 629 | 658 | 674 | 684 | 678 | 730 | 728 | 755 |
| **Lys** | **AAG** | 362 | 360 | 362 | 228 | 251 | 254 | 249 | 264 | 282 | 281 | 279 |
| **Phe** | **TTT** | 962 | 958 | 959 | 642 | 686 | 696 | 706 | 703 | 739 | 739 | 732 |
| **Phe** | **TTC** | 531 | 524 | 526 | 327 | 358 | 361 | 357 | 367 | 367 | 367 | 376 |
| **Pro** | **CCA** | 313 | 306 | 305 | 198 | 215 | 220 | 222 | 221 | 224 | 224 | 228 |
| **Pro** | **CCT** | 410 | 407 | 410 | 287 | 314 | 310 | 325 | 318 | 336 | 336 | 345 |
| **Pro** | **CCC** | 208 | 204 | 206 | 164 | 175 | 180 | 179 | 183 | 183 | 183 | 191 |
| **Pro** | **CCG** | 156 | 156 | 155 | 75 | 87 | 89 | 91 | 89 | 93 | 93 | 99 |
| **Ser** | **AGT** | 398 | 393 | 393 | 258 | 279 | 285 | 282 | 291 | 291 | 291 | 288 |
| **Ser** | **TCA** | 421 | 415 | 418 | 211 | 225 | 228 | 232 | 225 | 245 | 244 | 247 |
| **Ser** | **TCT** | 579 | 575 | 579 | 354 | 374 | 377 | 379 | 392 | 401 | 399 | 405 |
| **Ser** | **AGC** | 121 | 119 | 118 | 85 | 93 | 93 | 97 | 96 | 107 | 107 | 110 |
| **Ser** | **TCC** | 314 | 311 | 314 | 235 | 245 | 251 | 251 | 258 | 254 | 256 | 263 |
| **Ser** | **TCG** | 190 | 193 | 189 | 106 | 113 | 113 | 114 | 119 | 120 | 120 | 114 |
| **Thr** | **ACA** | 404 | 400 | 397 | 259 | 275 | 277 | 280 | 281 | 299 | 299 | 315 |
| **Thr** | **ACT** | 515 | 507 | 509 | 401 | 431 | 436 | 434 | 436 | 455 | 455 | 454 |
| **Thr** | **ACC** | 264 | 261 | 263 | 159 | 176 | 169 | 173 | 178 | 178 | 178 | 185 |
| **Thr** | **ACG** | 150 | 150 | 151 | 105 | 115 | 117 | 117 | 120 | 120 | 120 | 123 |
| **Tyr** | **TAT** | 784 | 776 | 771 | 505 | 527 | 530 | 543 | 543 | 567 | 567 | 575 |
| **Tyr** | **TAC** | 198 | 195 | 193 | 131 | 143 | 139 | 144 | 143 | 144 | 144 | 156 |
| **Val** | **GTA** | 512 | 509 | 510 | 383 | 410 | 410 | 415 | 414 | 443 | 442 | 449 |
| **Val** | **GTT** | 501 | 501 | 499 | 370 | 397 | 399 | 402 | 402 | 420 | 420 | 431 |
| **Val** | **GTC** | 177 | 178 | 180 | 127 | 134 | 137 | 134 | 139 | 144 | 144 | 144 |
| **Val** | **GTG** | 210 | 211 | 208 | 128 | 136 | 139 | 138 | 145 | 148 | 148 | 149 |
| **Stop** | **TAA** | 50 | 47 | 47 | 35 | 40 | 43 | 40 | 43 | 45 | 45 | 49 |
| **Stop** | **TGA** | 17 | 17 | 17 | 12 | 16 | 16 | 18 | 17 | 17 | 17 | 17 |
| **Stop** | **TAG** | 19 | 19 | 19 | 12 | 20 | 19 | 19 | 19 | 20 | 20 | 19 |
| **Met** | **ATG** | 607 | 599 | 602 | 380 | 406 | 413 | 424 | 419 | 457 | 457 | 469 |
| **Trp** | **TGG** | 461 | 452 | 457 | 308 | 326 | 327 | 337 | 328 | 347 | 347 | 348 |

**S2 Table. The relative synonymous codon usage (RSCU) in plastid genomes.**

| **Codon** | **Cotton** |  |  | **CV** | **Wheat** |  |  |  |  |  |  |  | **CV** |
| --- | --- | --- | --- | --- | --- | --- | --- | --- | --- | --- | --- | --- | --- |
|  | ***G. herbaceum*** | ***G. raimondii*** | ***G. hirsutum*** |  | ***T. urartu*** | ***T. boeoticum*** | ***T. monococcum*** | ***A. speltoides*** | ***A. tauschii*** | ***T. dicoccoides*** | ***T. turgidum*** | ***T. aestivum*** |  |
| **GCA** | 1.07 | 1.06 | 1.03 | 0.020 | 1.2 | 1.19 | 1.2 | 1.19 | 1.18 | 1.21 | 1.21 | 1.21 | 0.009 |
| **GCC** | 0.69 | 0.69 | 0.7 | 0.008 | 0.57 | 0.57 | 0.58 | 0.59 | 0.58 | 0.58 | 0.58 | 0.61 | 0.022 |
| **GCG** | 0.49 | 0.49 | 0.5 | 0.012 | 0.46 | 0.45 | 0.45 | 0.44 | 0.47 | 0.44 | 0.44 | 0.44 | 0.025 |
| **GCT** | 1.75 | 1.76 | 1.77 | 0.006 | 1.77 | 1.79 | 1.78 | 1.78 | 1.78 | 1.77 | 1.77 | 1.74 | 0.008 |
| **AGA** | 1.74 | 1.75 | 1.72 | 0.009 | 1.75 | 1.78 | 1.75 | 1.75 | 1.76 | 1.79 | 1.79 | 1.79 | 0.011 |
| **AGG** | 0.69 | 0.68 | 0.67 | 0.015 | 0.68 | 0.67 | 0.67 | 0.67 | 0.68 | 0.61 | 0.61 | 0.62 | 0.049 |
| **CGA** | 1.39 | 1.4 | 1.41 | 0.007 | 1.26 | 1.25 | 1.26 | 1.26 | 1.26 | 1.24 | 1.24 | 1.26 | 0.007 |
| **CGC** | 0.47 | 0.47 | 0.47 | 0.000 | 0.57 | 0.55 | 0.54 | 0.56 | 0.53 | 0.54 | 0.54 | 0.54 | 0.024 |
| **CGG** | 0.45 | 0.45 | 0.46 | 0.013 | 0.42 | 0.42 | 0.42 | 0.43 | 0.43 | 0.41 | 0.41 | 0.42 | 0.018 |
| **CGT** | 1.26 | 1.26 | 1.26 | 0.000 | 1.33 | 1.33 | 1.36 | 1.33 | 1.34 | 1.4 | 1.4 | 1.37 | 0.022 |
| **AAC** | 0.47 | 0.47 | 0.47 | 0.000 | 0.47 | 0.5 | 0.49 | 0.49 | 0.49 | 0.52 | 0.52 | 0.51 | 0.035 |
| **AAT** | 1.53 | 1.53 | 1.53 | 0.000 | 1.53 | 1.5 | 1.51 | 1.51 | 1.51 | 1.48 | 1.48 | 1.49 | 0.012 |
| **GAC** | 0.41 | 0.4 | 0.4 | 0.014 | 0.45 | 0.45 | 0.45 | 0.44 | 0.46 | 0.43 | 0.43 | 0.43 | 0.026 |
| **GAT** | 1.59 | 1.6 | 1.6 | 0.004 | 1.55 | 1.55 | 1.55 | 1.56 | 1.54 | 1.57 | 1.57 | 1.57 | 0.007 |
| **TGC** | 0.49 | 0.5 | 0.51 | 0.020 | 0.48 | 0.48 | 0.48 | 0.49 | 0.49 | 0.48 | 0.48 | 0.44 | 0.033 |
| **TGT** | 1.51 | 1.5 | 1.49 | 0.007 | 1.52 | 1.52 | 1.52 | 1.51 | 1.51 | 1.52 | 1.52 | 1.56 | 0.010 |
| **CAA** | 1.53 | 1.53 | 1.53 | 0.000 | 1.52 | 1.54 | 1.54 | 1.53 | 1.53 | 1.55 | 1.55 | 1.55 | 0.007 |
| **CAG** | 0.47 | 0.47 | 0.47 | 0.000 | 0.48 | 0.46 | 0.46 | 0.47 | 0.47 | 0.45 | 0.45 | 0.45 | 0.024 |
| **GAA** | 1.48 | 1.48 | 1.48 | 0.000 | 1.49 | 1.5 | 1.5 | 1.49 | 1.49 | 1.5 | 1.5 | 1.49 | 0.004 |
| **GAG** | 0.52 | 0.52 | 0.52 | 0.000 | 0.51 | 0.5 | 0.5 | 0.51 | 0.51 | 0.5 | 0.5 | 0.51 | 0.011 |
| **GGA** | 1.58 | 1.58 | 1.58 | 0.000 | 1.57 | 1.55 | 1.55 | 1.55 | 1.55 | 1.57 | 1.57 | 1.57 | 0.007 |
| **GGC** | 0.43 | 0.43 | 0.42 | 0.014 | 0.44 | 0.45 | 0.45 | 0.45 | 0.45 | 0.45 | 0.45 | 0.45 | 0.008 |
| **GGG** | 0.72 | 0.73 | 0.73 | 0.008 | 0.71 | 0.71 | 0.71 | 0.72 | 0.7 | 0.7 | 0.7 | 0.68 | 0.017 |
| **GGT** | 1.26 | 1.27 | 1.27 | 0.005 | 1.27 | 1.29 | 1.29 | 1.28 | 1.29 | 1.29 | 1.29 | 1.29 | 0.006 |
| **CAC** | 0.51 | 0.51 | 0.51 | 0.000 | 0.52 | 0.52 | 0.52 | 0.54 | 0.52 | 0.52 | 0.52 | 0.53 | 0.014 |
| **CAT** | 1.49 | 1.49 | 1.49 | 0.000 | 1.48 | 1.48 | 1.48 | 1.46 | 1.48 | 1.48 | 1.48 | 1.47 | 0.005 |
| **ATA** | 0.93 | 0.92 | 0.93 | 0.006 | 0.92 | 0.93 | 0.93 | 0.94 | 0.94 | 0.92 | 0.92 | 0.94 | 0.010 |
| **ATC** | 0.6 | 0.6 | 0.59 | 0.010 | 0.55 | 0.54 | 0.53 | 0.54 | 0.54 | 0.55 | 0.55 | 0.54 | 0.013 |
| **ATT** | 1.48 | 1.48 | 1.48 | 0.000 | 1.53 | 1.53 | 1.54 | 1.52 | 1.52 | 1.53 | 1.53 | 1.52 | 0.005 |
| **CTA** | 0.86 | 0.85 | 0.86 | 0.007 | 0.87 | 0.86 | 0.85 | 0.86 | 0.86 | 0.86 | 0.86 | 0.88 | 0.010 |
| **CTC** | 0.41 | 0.4 | 0.41 | 0.014 | 0.43 | 0.41 | 0.42 | 0.42 | 0.42 | 0.41 | 0.41 | 0.42 | 0.017 |
| **CTG** | 0.43 | 0.42 | 0.41 | 0.024 | 0.3 | 0.31 | 0.31 | 0.3 | 0.31 | 0.31 | 0.31 | 0.3 | 0.017 |
| **CTT** | 1.24 | 1.25 | 1.25 | 0.005 | 1.3 | 1.28 | 1.27 | 1.28 | 1.27 | 1.27 | 1.27 | 1.26 | 0.009 |
| **TTA** | 1.83 | 1.83 | 1.83 | 0.000 | 1.99 | 2.05 | 2.05 | 2.05 | 2.04 | 2.06 | 2.06 | 2.06 | 0.011 |
| **TTG** | 1.23 | 1.24 | 1.24 | 0.005 | 1.12 | 1.09 | 1.09 | 1.09 | 1.09 | 1.08 | 1.08 | 1.08 | 0.012 |
| **AAA** | 1.49 | 1.48 | 1.48 | 0.004 | 1.47 | 1.45 | 1.45 | 1.47 | 1.44 | 1.44 | 1.44 | 1.46 | 0.009 |
| **AAG** | 0.51 | 0.52 | 0.52 | 0.011 | 0.53 | 0.55 | 0.55 | 0.53 | 0.56 | 0.56 | 0.56 | 0.54 | 0.023 |
| **TTC** | 0.71 | 0.71 | 0.71 | 0.000 | 0.67 | 0.69 | 0.68 | 0.67 | 0.69 | 0.66 | 0.66 | 0.68 | 0.018 |
| **TTT** | 1.29 | 1.29 | 1.29 | 0.000 | 1.33 | 1.31 | 1.32 | 1.33 | 1.31 | 1.34 | 1.34 | 1.32 | 0.009 |
| **CCA** | 1.15 | 1.14 | 1.13 | 0.009 | 1.09 | 1.09 | 1.1 | 1.09 | 1.09 | 1.07 | 1.07 | 1.06 | 0.013 |
| **CCC** | 0.77 | 0.76 | 0.77 | 0.008 | 0.91 | 0.88 | 0.9 | 0.88 | 0.9 | 0.88 | 0.88 | 0.89 | 0.013 |
| **CCG** | 0.57 | 0.58 | 0.58 | 0.010 | 0.41 | 0.44 | 0.45 | 0.45 | 0.44 | 0.44 | 0.44 | 0.46 | 0.033 |
| **CCT** | 1.51 | 1.52 | 1.52 | 0.004 | 1.59 | 1.59 | 1.55 | 1.59 | 1.57 | 1.61 | 1.61 | 1.6 | 0.013 |
| **AGC** | 0.36 | 0.36 | 0.35 | 0.016 | 0.41 | 0.42 | 0.41 | 0.43 | 0.42 | 0.45 | 0.45 | 0.46 | 0.045 |
| **AGT** | 1.18 | 1.18 | 1.17 | 0.005 | 1.24 | 1.26 | 1.27 | 1.25 | 1.26 | 1.23 | 1.23 | 1.21 | 0.016 |
| **TCA** | 1.25 | 1.24 | 1.25 | 0.005 | 1.01 | 1.02 | 1.02 | 1.03 | 0.98 | 1.04 | 1.03 | 1.04 | 0.019 |
| **TCC** | 0.93 | 0.93 | 0.94 | 0.006 | 1.13 | 1.11 | 1.12 | 1.11 | 1.12 | 1.07 | 1.08 | 1.11 | 0.019 |
| **TCG** | 0.56 | 0.58 | 0.56 | 0.020 | 0.51 | 0.51 | 0.5 | 0.5 | 0.52 | 0.51 | 0.51 | 0.48 | 0.024 |
| **TCT** | 1.72 | 1.72 | 1.73 | 0.003 | 1.7 | 1.69 | 1.68 | 1.68 | 1.7 | 1.7 | 1.69 | 1.7 | 0.005 |
| **ACA** | 1.21 | 1.21 | 1.2 | 0.005 | 1.12 | 1.1 | 1.11 | 1.12 | 1.11 | 1.14 | 1.14 | 1.17 | 0.020 |
| **ACC** | 0.79 | 0.79 | 0.8 | 0.007 | 0.69 | 0.71 | 0.68 | 0.69 | 0.7 | 0.68 | 0.68 | 0.69 | 0.015 |
| **ACG** | 0.45 | 0.46 | 0.46 | 0.013 | 0.45 | 0.46 | 0.47 | 0.47 | 0.47 | 0.46 | 0.46 | 0.46 | 0.015 |
| **ACT** | 1.55 | 1.54 | 1.54 | 0.004 | 1.74 | 1.73 | 1.75 | 1.73 | 1.72 | 1.73 | 1.73 | 1.69 | 0.010 |
| **TAC** | 0.4 | 0.4 | 0.4 | 0.000 | 0.41 | 0.43 | 0.42 | 0.42 | 0.42 | 0.41 | 0.41 | 0.43 | 0.020 |
| **TAT** | 1.6 | 1.6 | 1.6 | 0.000 | 1.59 | 1.57 | 1.58 | 1.58 | 1.58 | 1.59 | 1.59 | 1.57 | 0.005 |
| **GTA** | 1.46 | 1.46 | 1.46 | 0.000 | 1.52 | 1.52 | 1.51 | 1.52 | 1.51 | 1.53 | 1.53 | 1.53 | 0.005 |
| **GTC** | 0.51 | 0.51 | 0.52 | 0.011 | 0.5 | 0.5 | 0.51 | 0.49 | 0.51 | 0.5 | 0.5 | 0.49 | 0.015 |
| **GTG** | 0.6 | 0.6 | 0.6 | 0.000 | 0.51 | 0.51 | 0.51 | 0.51 | 0.53 | 0.51 | 0.51 | 0.51 | 0.014 |
| **GTT** | 1.43 | 1.43 | 1.43 | 0.000 | 1.47 | 1.47 | 1.47 | 1.48 | 1.46 | 1.45 | 1.46 | 1.47 | 0.006 |

**S3 Table. Indices of synonymous codon usage bias in the plastid genomes.**

| **Species** | **CAI** | **CBI** | **Fop** | **Nc** |
| --- | --- | --- | --- | --- |
| ***G. herbaceum*** | 0.166 | -0.102 | 0.353 | 50.53 |
| ***G. raimondii*** | 0.165 | -0.103 | 0.353 | 50.48 |
| ***G. hirsutum*** | 0.166 | -0.103 | 0.353 | 50.49 |
| ***T. urartu*** | 0.168 | -0.091 | 0.358 | 49.8 |
| ***T. boeoticum*** | 0.168 | -0.09 | 0.358 | 49.78 |
| ***T. monococcum*** | 0.168 | -0.09 | 0.358 | 49.76 |
| ***A. speltoides*** | 0.167 | -0.092 | 0.357 | 49.82 |
| ***A. tauschii*** | 0.167 | -0.09 | 0.358 | 49.92 |
| ***T. dicoccoides*** | 0.168 | -0.091 | 0.357 | 49.59 |
| ***T. turgidum*** | 0.168 | -0.091 | 0.357 | 49.6 |
| ***T. aestivum*** | 0.167 | -0.091 | 0.357 | 49.67 |

**S4 Table. The statistical analysis of SCUB frequencies.**

| **Taxomony** | **Species** | **SCUB based on amino acids** | | |  | **SCUB based on NNA/T and NNC/G** | | |
| --- | --- | --- | --- | --- | --- | --- | --- | --- |
|  |  | **Mean** | **CV** | ***P* value ^a^** |  | **NNA/T** | **NNC/G** | ***P* value ^b^** |
| **cotton** | *G. herbaceum* | 0.378 | 0.243 | 7.42E-16 |  | 18288 | 8099 | < 0.001 |
|  | *G. raimondii* | 0.378 | 0.243 | 7.25E-16 |  | 18108 | 8010 | < 0.001 |
|  | *G. hirsutum* | 0.378 | 0.243 | 7.76E-16 |  | 18118 | 8019 | < 0.001 |
|  |  |  |  |  |  |  |  |  |
| **wheat** | *T. urartu* | 0.367 | 0.225 | 9.39E-17 |  | 12024 | 5164 | < 0.001 |
|  | *T. boeoticum* | 0.369 | 0.226 | 1.13E-16 |  | 12796 | 5506 | < 0.001 |
|  | *T. monococcum* | 0.368 | 0.231 | 1.56E-16 |  | 12921 | 5547 | < 0.001 |
|  | *A. speltoides* | 0.368 | 0.223 | 9.12E-17 |  | 13090 | 5629 | < 0.001 |
|  | *A. tauschii* | 0.372 | 0.228 | 1.64E-16 |  | 13102 | 5680 | < 0.001 |
|  | *T. dicoccoides* | 0.363 | 0.227 | 7.97E-17 |  | 13717 | 5850 | < 0.001 |
|  | *T. turgidum* | 0.363 | 0.227 | 8.51E-17 |  | 13709 | 5852 | < 0.001 |
|  | *T. aestivum* | 0.378 | 0.243 | 1.70E-16 |  | 14002 | 5980 | < 0.001 |

**S5 Table. Correlation coefficients between the SCUB frequencies associated with each amino acid encoded by genes harboured by the chloroplast genomes.**

| **Plants** | **Ploidy** | **Species** | ***r* values** |  |  |  |  |  |  |
| --- | --- | --- | --- | --- | --- | --- | --- | --- | --- |
| **Cotton** |  |  | *G. herbaceum* | *G. raimondii* |  |  |  |  |  |
|  | AA | *G. herbaceum* |  |  |  |  |  |  |  |
|  | *DD* | *G. raimondii* | 0.9994 |  |  |  |  |  |  |
|  | *AADD* | *G. hirsutum* | 0.9987 | 0.9989 |  |  |  |  |  |
|  |  |  |  |  |  |  |  |  |  |
| **Wheat** |  |  | *T. urartu* | *T. boeoticum* | *T. monococcum* | *A. speltoides* | *A. tauschii* | *T. dicoccoides* | *T. turgidum* |
|  | *AA* | *T. urartu* |  |  |  |  |  |  |  |
|  | *AA* | *T. boeoticum* | 0.9928 |  |  |  |  |  |  |
|  | *AA* | *T. monococcum* | 0.9945 | 0.9981 |  |  |  |  |  |
|  | *BB* | *A. speltoides* | 0.9943 | 0.9937 | 0.9946 |  |  |  |  |
|  | *DD* | *A. tauschii* | 0.9937 | 0.9984 | 0.9985 | 0.9926 |  |  |  |
|  | *AABB* | *T. dicoccoides* | 0.9817 | 0.9907 | 0.9923 | 0.9864 | 0.9920 |  |  |
|  | *AABB* | *T. turgidum* | 0.9817 | 0.9904 | 0.9919 | 0.9863 | 0.9918 | 0.9999 |  |
|  | *AABBDD* | *T. aestivum* | 0.9807 | 0.9895 | 0.9898 | 0.9869 | 0.9882 | 0.9907 | 0.9906 |

**S6 Table. The comparison on the ratios of NNC/NNG to NNA/NNT with the ratios of C and G to A and T in the gene body and whole genome sequences.**

| **Taxomony** | **Species** | **CDS** | **Gene** |  |  | **Genome** |  |
| --- | --- | --- | --- | --- | --- | --- | --- |
|  |  | **NNC/G to NNA/T** | **Ratio of CG to AT** | **P value** |  | **Ratio of CG to AT** | **P value** |
| **cotton** | *G. herbaceum* | 0.443 | 0.670 | 5.23E-65 |  | 0.595 | 3.86E-95 |
|  | *G. raimondii* | 0.442 | 0.688 | 6.44E-74 |  | 0.595 | 5.19E-95 |
|  | *G. hirsutum* | 0.443 | 0.691 | 3.59E-75 |  | 0.594 | 4.71E-93 |
|  |  |  |  |  |  |  |  |
| **wheat** | *T. urartu* | 0.429 | 0.686 | 2.29E-71 |  | 0.597 | 1.44E-77 |
|  | *T. boeoticum* | 0.430 | 0.687 | 4.64E-73 |  | 0.600 | 3.16E-83 |
|  | *T. monococcum* | 0.429 | 0.676 | 4.79E-69 |  | 0.597 | 1.39E-82 |
|  | *A. speltoides* | 0.430 | 0.687 | 2.40E-74 |  | 0.594 | 4.85E-80 |
|  | *A. tauschii* | 0.434 | 0.683 | 1.88E-69 |  | 0.594 | 1.04E-76 |
|  | *T. dicoccoides* | 0.426 | 0.690 | 2.63E-79 |  | 0.621 | 1.57E-114 |
|  | *T. turgidum* | 0.427 | 0.691 | 3.27E-79 |  | 0.621 | 1.70E-114 |
|  | *T. aestivum* | 0.427 | 0.690 | 2.72E-79 |  | 0.621 | 6.70E-116 |

**S7 Table. The statistical analysis of the effect of the nucleotide at the second** potion and the first position of the downstream codon on the ratio of G and C at the third position.

| **Species** | **NAG/NAC** | **NCG/NCC** | **NGG/NGC** | **NTG/NTC** |  | **NC|A/NG|A** | **NC|C/NG|C** | **NC|G/NG|G** | **NC|T/NG|T** |
| --- | --- | --- | --- | --- | --- | --- | --- | --- | --- |
| ***G. herbaceum*** | 0.154 | 1.550E-18 | 1.509E-46 | 8.647E-06 |  | 0.001 | 1.168E-04 | 7.486E-24 | 0.005 |
| ***G. raimondii*** | 0.115 | 1.835E-17 | 3.209E-45 | 8.565E-06 |  | 3.982E-4 | 8.100E-05 | 2.523E-24 | 0.006 |
| ***G. hirsutum*** | 0.090 | 3.338E-18 | 3.066E-47 | 1.508E-05 |  | 0.001 | 7.035E-05 | 2.159E-25 | 0.007 |
|  |  |  |  |  |  |  |  |  |  |
| ***T. urartu*** | 0.051 | 2.259E-19 | 1.707E-26 | 0.012 |  | 0.007 | 6.358E-05 | 8.905E-21 | 1.455E-07 |
| ***T. boeoticum*** | 0.168 | 9.082E-20 | 1.000E-27 | 0.025 |  | 0.035 | 1.152E-04 | 1.701E-21 | 1.143E-08 |
| ***T. monococcum*** | 0.086 | 6.008E-20 | 3.371E-28 | 0.011 |  | 0.013 | 3.034E-04 | 1.559E-21 | 1.827E-08 |
| ***A. speltoides*** | 0.119 | 2.942E-20 | 8.610E-29 | 0.006 |  | 0.017 | 4.147E-04 | 1.365E-21 | 5.004E-08 |
| ***A. tauschii*** | 0.066 | 5.721E-20 | 4.209E-28 | 0.014 |  | 0.021 | 2.803E-04 | 7.411E-22 | 1.519E-08 |
| ***T. dicoccoides*** | 0.026 | 2.631E-20 | 1.053E-26 | 0.001 |  | 0.008 | 0.001 | 2.363E-23 | 2.099E-08 |
| ***T. turgidum*** | 0.028 | 1.664E-20 | 1.053E-26 | 0.001 |  | 0.009 | 0.001 | 4.026E-23 | 2.099E-08 |
| ***T. aestivum*** | 0.075 | 2.641E-23 | 2.246E-26 | 0.001 |  | 0.033 | 5.377E-05 | 2.055E-23 | 1.126E-08 |

**S8 Table. The statistical analysis of the association between DNA-methylation and SCUB.**

| **Species** | **NCG/NCC** |  |  |  | **NC|G/NG|G** |  |  |
| --- | --- | --- | --- | --- | --- | --- | --- |
|  | **NAG/NAC** | **NGG/NGC** | **NTG/NTC** |  | **NC|A/NG|A** | **NC|C/NG|C** | **NC|T/NG|T** |
| ***G. herbaceum*** | 2.731E-13 | 3.431E-14 | 4.272E-14 |  | 1.241E-06 | 3.804E-14 | 5.117E-14 |
| ***G. raimondii*** | 5.758E-13 | 3.575E-14 | 4.652E-14 |  | 1.107E-06 | 3.916E-14 | 3.777E-14 |
| ***G. hirsutum*** | 1.408E-13 | 3.704E-14 | 3.798E-14 |  | 1.631E-07 | 4.517E-14 | 5.084E-14 |
|  |  |  |  |  |  |  |  |
| ***T. urartu*** | 5.160E-14 | 3.834E-14 | 3.145E-14 |  | 3.251E-06 | 3.905E-14 | 3.264E-14 |
| ***T. boeoticum*** | 1.193E-13 | 2.787E-14 | 5.112E-14 |  | 1.578E-07 | 3.316E-14 | 4.038E-14 |
| ***T. monococcum*** | 4.052E-14 | 2.930E-14 | 3.001E-14 |  | 7.396E-07 | 3.249E-14 | 4.364E-14 |
| ***A. speltoides*** | 6.457E-14 | 3.421E-14 | 4.381E-14 |  | 4.461E-07 | 4.341E-14 | 3.315E-14 |
| ***A. tauschii*** | 3.229E-14 | 3.321E-14 | 3.460E-14 |  | 2.399E-07 | 3.435E-14 | 2.878E-14 |
| ***T. dicoccoides*** | 4.436E-14 | 2.805E-14 | 5.069E-14 |  | 2.439E-07 | 4.008E-14 | 4.944E-14 |
| ***T. turgidum*** | 2.722E-14 | 3.909E-14 | 3.778E-14 |  | 2.498E-07 | 3.173E-14 | 3.924E-14 |
| ***T. aestivum*** | 4.706E-14 | 3.038E-14 | 4.836E-14 |  | 3.227E-08 | 3.660E-14 | 3.483E-14 |

**S9 Table. The comparison between the NNG/NNC with C and other nucleotide** at the second positon in amino acids encoded by C-and G-ending SCs.

| **Species** | **Ala** | **Pro** | **Ser** | **Thr** |  | **Arg** | **Gly** | **Leu** | **Val** |  | **P value** |
| --- | --- | --- | --- | --- | --- | --- | --- | --- | --- | --- | --- |
|  | **NCG/NCC** | **NCG/NCC** | **NCG/NCC** | **NCG/NCC** |  | **NGG/NGC** | **NGG/NGC** | **NTG/NTC** | **NTG/NTC** |  |  |
| ***G. herbaceum*** | 0.707 | 0.750 | 0.605 | 0.568 |  | 0.952 | 1.670 | 1.053 | 1.186 |  | 0.007 |
| ***G. raimondii*** | 0.703 | 0.765 | 0.621 | 0.575 |  | 0.944 | 1.677 | 1.054 | 1.185 |  | 0.008 |
| ***G. hirsutum*** | 0.714 | 0.752 | 0.602 | 0.574 |  | 0.984 | 1.723 | 1.005 | 1.156 |  | 0.010 |
|  |  |  |  |  |  |  |  |  |  |  |  |
| ***T. urartu*** | 0.808 | 0.457 | 0.451 | 0.660 |  | 0.729 | 1.608 | 0.697 | 1.008 |  | 0.059 |
| ***T. boeoticum*** | 0.788 | 0.497 | 0.461 | 0.653 |  | 0.762 | 1.586 | 0.757 | 1.015 |  | 0.042 |
| ***T. monococcum*** | 0.774 | 0.494 | 0.450 | 0.692 |  | 0.780 | 1.582 | 0.730 | 1.015 |  | 0.045 |
| ***A. speltoides*** | 0.756 | 0.508 | 0.454 | 0.676 |  | 0.781 | 1.596 | 0.723 | 1.030 |  | 0.043 |
| ***A. tauschii*** | 0.806 | 0.486 | 0.461 | 0.674 |  | 0.804 | 1.561 | 0.736 | 1.043 |  | 0.040 |
| ***T. dicoccoides*** | 0.744 | 0.508 | 0.472 | 0.674 |  | 0.764 | 1.558 | 0.752 | 1.028 |  | 0.038 |
| ***T. turgidum*** | 0.744 | 0.508 | 0.469 | 0.674 |  | 0.764 | 1.558 | 0.752 | 1.028 |  | 0.038 |
| ***T. aestivum*** | 0.708 | 0.518 | 0.433 | 0.665 |  | 0.780 | 1.491 | 0.709 | 1.035 |  | 0.033 |

**S10 Table. The statistical analysis of the difference between NNC and NNC of amino acids encoded by C- and G-ending SCs.**

| **Species** | **Ala** | **Pro** | **Ser** | **Thr** |  | **Arg** | **Gly** | **Leu** | **Val** |
| --- | --- | --- | --- | --- | --- | --- | --- | --- | --- |
|  | **NCG/NCC** | **NCG/NCC** | **NCG/NCC** | **NCG/NCC** |  | **NGG/NGC** | **NGG/NGC** | **NTG/NTC** | **NTG/NTC** |
| ***G. herbaceum*** | 3.99E-04 | 0.006 | 3.33E-08 | 2.11E-08 |  | 0.702 | 8.64E-09 | 0.613 | 0.093 |
| ***G. raimondii*** | 3.62E-04 | 0.011 | 1.47E-07 | 4.37E-08 |  | 0.652 | 7.58E-09 | 0.607 | 0.094 |
| ***G. hirsutum*** | 0.001 | 0.007 | 2.50E-08 | 3.70E-08 |  | 0.899 | 1.43E-09 | 0.959 | 0.155 |
|  |  |  |  |  |  |  |  |  |  |
| ***T. urartu*** | 0.074 | 0.000 | 2.83E-12 | 0.001 |  | 0.044 | 6.65E-06 | 0.008 | 0.950 |
| ***T. boeoticum*** | 0.042 | 0.000 | 3.03E-12 | 3.49E-04 |  | 0.072 | 7.14E-06 | 0.033 | 0.903 |
| ***T. monococcum*** | 0.028 | 0.000 | 4.72E-13 | 0.002 |  | 0.099 | 7.53E-06 | 0.015 | 0.904 |
| ***A. speltoides*** | 0.016 | 0.000 | 7.45E-13 | 0.001 |  | 0.093 | 3.82E-06 | 0.012 | 0.808 |
| ***A. tauschii*** | 0.060 | 0.000 | 8.13E-13 | 0.001 |  | 0.140 | 1.14E-05 | 0.016 | 0.722 |
| ***T. dicoccoides*** | 0.009 | 0.000 | 4.24E-12 | 0.001 |  | 0.068 | 8.34E-06 | 0.024 | 0.815 |
| ***T. turgidum*** | 0.009 | 0.000 | 2.32E-12 | 0.001 |  | 0.068 | 8.34E-06 | 0.024 | 0.815 |
| ***T. aestivum*** | 0.002 | 0.000 | 1.67E-14 | 4.11E-04 |  | 0.085 | 4.71E-05 | 0.006 | 0.770 |

**S11 Table. The correlation coefficients of SCs in different PCs.**

|  | **Cotton** |  |  | **Wheat** |  |  |
| --- | --- | --- | --- | --- | --- | --- |
| **Codon** | **PC1** | **PC2** |  | **PC1** | **PC2** | **PC3** |
| **GCA** | -0.908 | -0.420 |  | -0.387 | -0.776 | -0.354 |
| **AGA** | -0.815 | -0.579 |  | -0.885 | 0.406 | -0.014 |
| **CGA** | 0.944 | -0.329 |  | -0.257 | 0.616 | 0.580 |
| **CAA** | 0.331 | 0.944 |  | -0.788 | 0.384 | -0.084 |
| **GAA** | -1.000 | -0.016 |  | 0.080 | -0.950 | -0.202 |
| **GGA** | -0.046 | 0.999 |  | -0.395 | -0.816 | 0.300 |
| **ATA** | 0.556 | 0.831 |  | 0.014 | 0.595 | 0.720 |
| **CTA** | -0.438 | 0.899 |  | -0.318 | -0.617 | 0.484 |
| **TTA** | 0.032 | 0.999 |  | -0.771 | 0.606 | -0.068 |
| **AAA** | -0.974 | 0.225 |  | -0.851 | -0.466 | 0.050 |
| **CCA** | -0.956 | 0.295 |  | 0.620 | 0.668 | 0.121 |
| **TCA** | 0.372 | 0.928 |  | -0.544 | -0.349 | -0.405 |
| **ACA** | -0.855 | -0.518 |  | -0.860 | -0.350 | 0.310 |
| **GTA** | 0.949 | -0.317 |  | -0.721 | -0.372 | -0.445 |
| **GCT** | 0.977 | -0.214 |  | 0.827 | -0.057 | -0.525 |
| **CGT** | 0.216 | 0.976 |  | -0.860 | 0.202 | -0.340 |
| **AAT** | 0.996 | 0.088 |  | 0.527 | 0.033 | 0.625 |
| **GAT** | 0.744 | -0.669 |  | 0.261 | -0.047 | 0.052 |
| **TGT** | -0.800 | -0.600 |  | -0.353 | -0.400 | 0.360 |
| **GGT** | 1.000 | -0.031 |  | -0.613 | -0.001 | 0.488 |
| **CAT** | -0.134 | 0.991 |  | 0.722 | -0.571 | 0.157 |
| **ATT** | 0.963 | -0.268 |  | 0.422 | 0.238 | -0.213 |
| **CTT** | 0.281 | -0.960 |  | 0.692 | -0.693 | -0.065 |
| **TTT** | 0.882 | -0.471 |  | 0.306 | 0.245 | -0.828 |
| **CCT** | 0.978 | 0.211 |  | -0.639 | 0.227 | 0.135 |
| **AGT** | -0.952 | 0.305 |  | 0.746 | 0.584 | -0.255 |
| **TCT** | 0.987 | 0.164 |  | 0.469 | 0.131 | -0.190 |
| **ACT** | -0.395 | 0.919 |  | 0.631 | 0.273 | -0.577 |
| **TAT** | -0.872 | -0.490 |  | 0.427 | -0.777 | -0.167 |
| **GTT** | 0.528 | -0.849 |  | 0.076 | 0.062 | 0.206 |
| **GCC** | 0.725 | 0.689 |  | -0.803 | -0.253 | 0.516 |
| **CGC** | -0.908 | 0.418 |  | 0.553 | -0.371 | 0.369 |
| **AAC** | -0.063 | 0.998 |  | -0.922 | 0.221 | -0.260 |
| **GAC** | -0.512 | 0.859 |  | 0.964 | 0.202 | 0.159 |
| **TGC** | 0.999 | 0.054 |  | 0.831 | 0.194 | -0.478 |
| **GGC** | -0.870 | -0.492 |  | -0.652 | -0.169 | 0.703 |
| **CAC** | -0.992 | -0.123 |  | 0.558 | -0.283 | 0.663 |
| **ATC** | -0.577 | -0.817 |  | 0.239 | -0.107 | -0.483 |
| **CTC** | -0.277 | 0.961 |  | 0.581 | -0.105 | 0.499 |
| **TTC** | 0.027 | 1.000 |  | 0.646 | 0.657 | 0.150 |
| **CCC** | -0.003 | 1.000 |  | 0.524 | 0.491 | 0.524 |
| **AGC** | -0.996 | -0.089 |  | -0.966 | -0.062 | -0.119 |
| **TCC** | 0.899 | 0.437 |  | 0.865 | 0.148 | 0.321 |
| **ACC** | 0.782 | 0.624 |  | 0.409 | 0.422 | 0.383 |
| **TAC** | -0.980 | -0.199 |  | 0.196 | 0.117 | 0.859 |
| **GTC** | 0.992 | -0.125 |  | 0.348 | 0.021 | -0.588 |
| **GCG** | 0.680 | 0.733 |  | 0.887 | -0.042 | 0.101 |
| **AGG** | -0.993 | 0.120 |  | 0.816 | 0.415 | 0.401 |
| **CGG** | 0.638 | 0.770 |  | 0.054 | 0.703 | 0.512 |
| **CAG** | 0.380 | -0.925 |  | 0.924 | -0.162 | 0.246 |
| **GAG** | 0.236 | 0.972 |  | 0.287 | -0.879 | 0.279 |
| **GGG** | 0.998 | 0.065 |  | 0.887 | -0.218 | -0.117 |
| **CTG** | -0.994 | -0.113 |  | 0.080 | 0.771 | -0.567 |
| **TTG** | 0.490 | -0.871 |  | 0.652 | -0.686 | -0.006 |
| **AAG** | 1.000 | -0.003 |  | -0.711 | 0.242 | -0.496 |
| **CCG** | 0.283 | -0.959 |  | -0.655 | 0.627 | 0.287 |
| **TCG** | 0.141 | -0.990 |  | 0.679 | 0.294 | -0.567 |
| **ACG** | 0.987 | -0.161 |  | 0.411 | 0.868 | 0.187 |
| **GTG** | -0.013 | -1.000 |  | -0.072 | 0.364 | -0.341 |


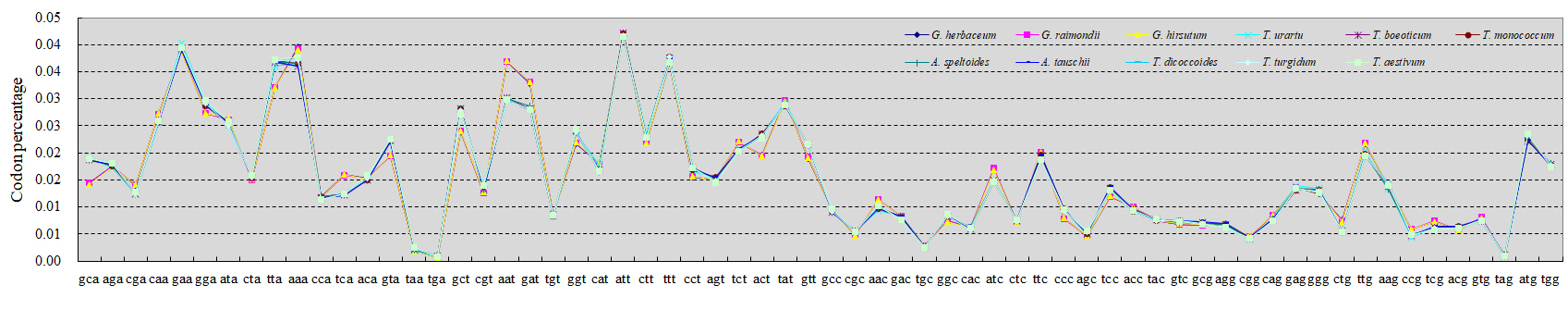


**S1 Fig**


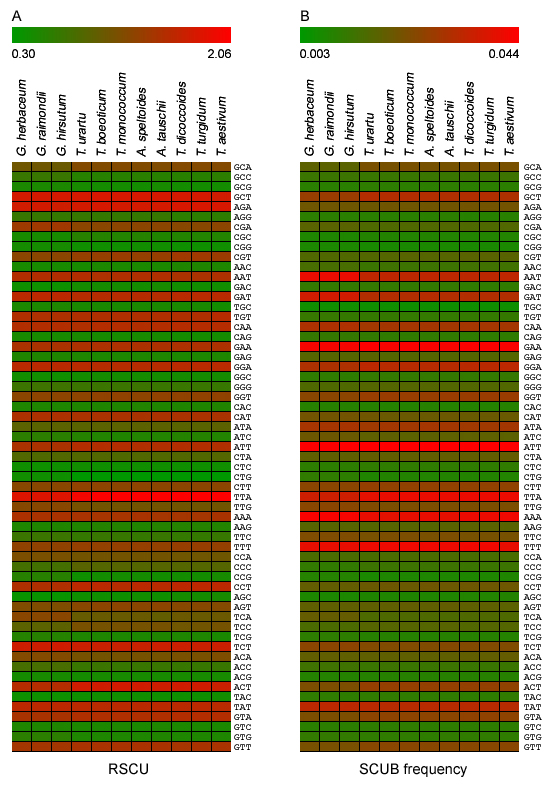


**S2 Fig**


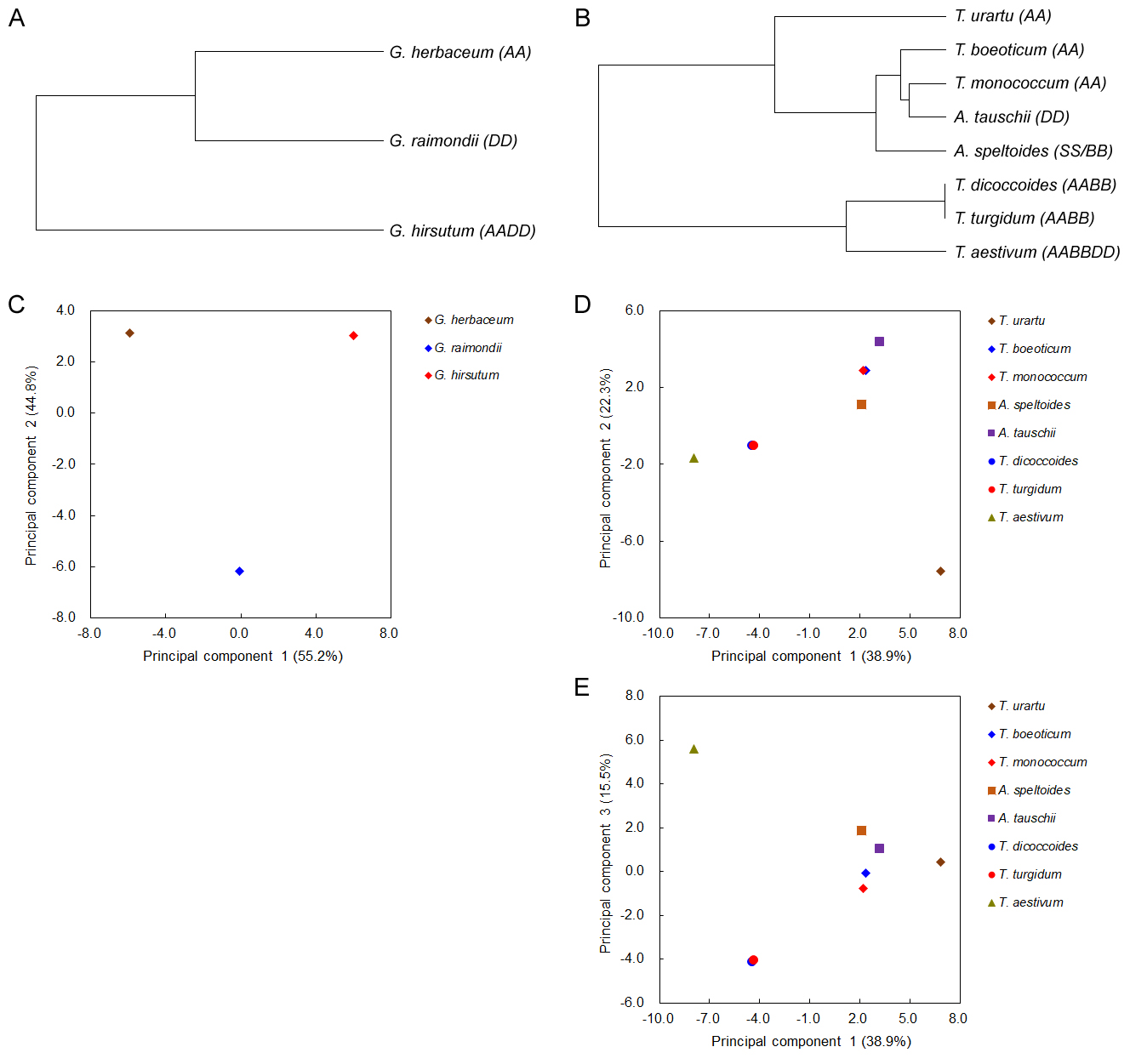


**S3 Fig**


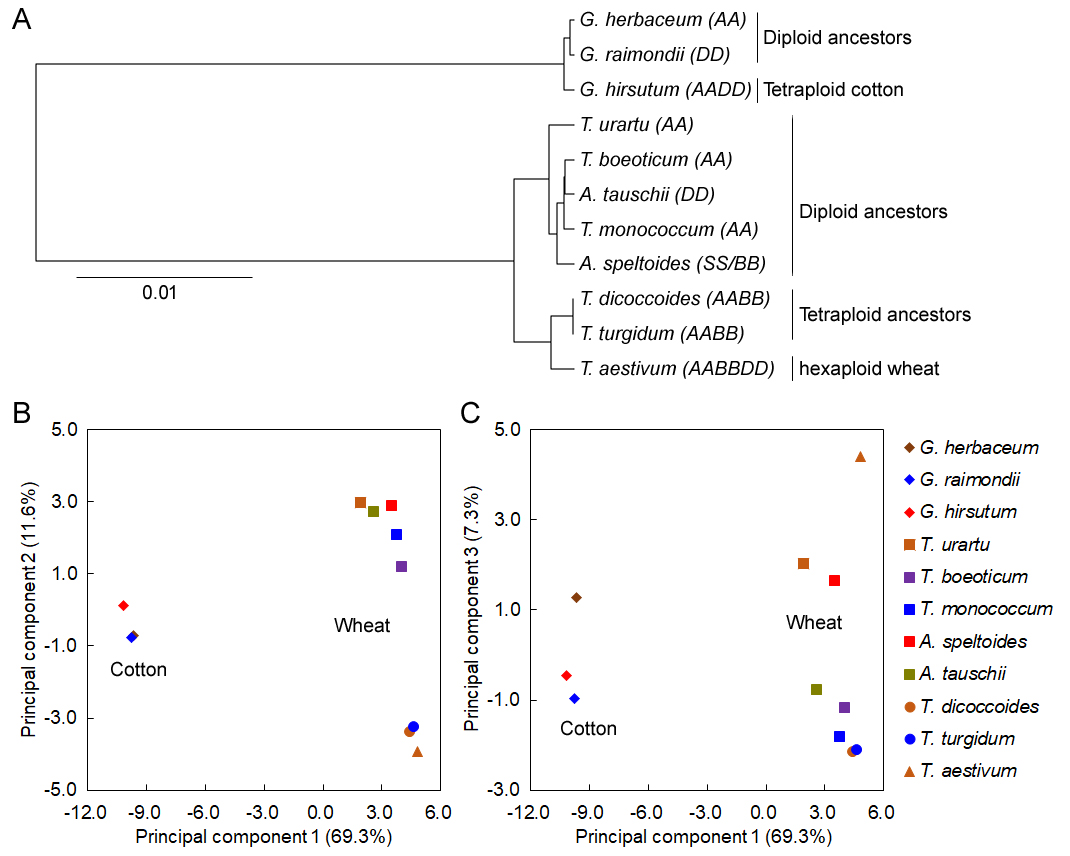


**S4 Fig**
